# Synthesis and Properties of [3]Rotaxanes with Two Oligo(*para‐*phenylene) Axles

**DOI:** 10.1002/chem.202500522

**Published:** 2025-04-14

**Authors:** Misuzu Ohta, Ayano Okuda, Kayori Takahashi, Shoichi Hosoya, Yusuke Yoshigoe, Shinichi Saito

**Affiliations:** ^1^ Department of Chemistry Tokyo University of Science 1-3, Kagurazaka, Shinjuku Tokyo 162-8601 Japan; ^2^ National Metrology Institute of Japan (NMIJ) National Institute of Advanced Industrial Science and Technology (AIST) Tsukuba Central 3, 1–1-1 Umezono, Tsukuba Ibaraki 305-8563 Japan; ^3^ Ochanomizu Research Facility, Bioscience Center Research Infrastructure Management Center Institute of Science Tokyo 1-5-45 Yushima, Bunkyo-ku Tokyo 113-8510 Japan

**Keywords:** [3]rotaxane, oligo(*para*-phenylene), aggregation, biaryl coupling

## Abstract

Oligo(*para*‐phenylene) (PPn) is a π‐conjugated compound where phenylene units are directly connected at the 1,4‐positions. Utilizing the catalytic activity of the macrocyclic dihydrodibenzophenanthroline‐Ni complex, we succeeded in the synthesis of [3]‐ and [2]rotaxanes with PPn axles by biaryl coupling. The size of the macrocycle was critical to synthesize [3]rotaxanes, which consist of two oligo(*para*‐phenylene) axles, in good yields. The relationship between the length of the PPn structure on the ^1^H NMR spectra of the rotaxanes was studied. The aggregation of a PP8 derivative was suppressed by the presence of the macrocycle in the rotaxanes. A [3]rotaxane could be considered as a model compound of an aggregated PPn, and the photophysical studies disclosed the influence of the aggregation on the properties of PPn.

## Graphical abstract

[3]‐ and [2]rotaxanes with oligo(*para*‐phenylene) axles were synthesized by Ni‐mediated biaryl coupling. [3]Rotaxanes were synthesized in good yields when a larger macrocyclic Ni complex was employed. The spectroscopic properties and the aggregation behavior of octa(*para*‐phenylene) derivatives were examined. The aggregated/non‐aggregated state could be controlled by utilizing the interlocked structure.

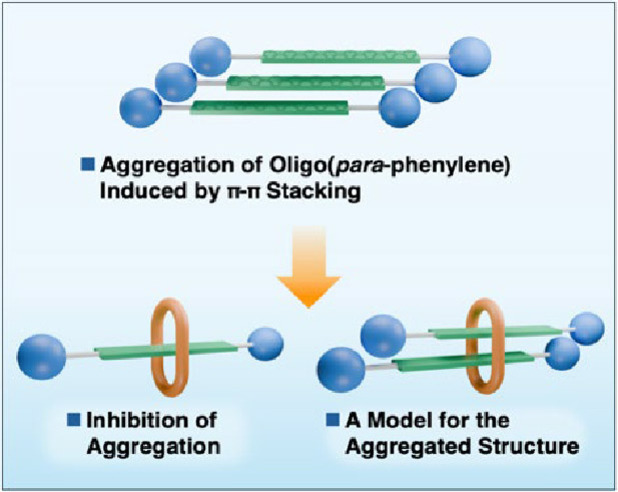

## Introduction

Oligo(*para‐*phenylene) (PPn) is a π‐conjugated compound which is composed of a defined number of 1,4‐connected phenylene moieties.[^1^, ^2^, ^3^, ^4^, ^5^] PPn would be a model compound for poly(*para‐*phenylene) which has been studied extensively due to its unique electronic and optical properties, especially as materials for organic light‐emitting diodes (OLEDs). Though long PPn is a very attractive compound because of its extended π‐system, the synthesis of longer PPn with six or more phenylene moieties (n≥6), is difficult because of its low solubility.[^6^, ^7^] The intermolecular π‐π interaction would induce the aggregation of the PPn, thus reducing the solubility. As discrete PPn, unsubstituted PP9 was synthesized as an insoluble compound^[6]^ and some soluble PP8 derivatives with substituents at the terminal position[^8^, ^9^, ^10^] have been reported.

**Scheme 1:**
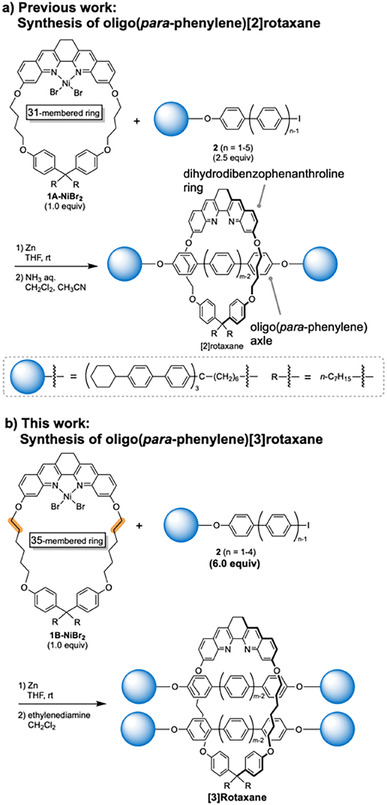
Synthesis of oligo(*para‐*phenylene)rotaxanes by biaryl coupling.

We recently reported the synthesis of [2]rotaxanes with oligo(*para‐*phenylene) axle by biaryl coupling mediated by macrocyclic dihydrodibenzophenanthroline‐Ni complex (Scheme 1a).[^11^, ^12^, ^13^] The reaction proceeded under mild conditions, and oligo(*para‐*phenylene) [2]rotaxanes were isolated in up to 82 % yield.[^14^, ^15^, ^16^, ^17^, ^18^, ^19^, ^20^] Interestingly, the solubility of the [2]rotaxanes, including a PP10 derivative, was very high compared to the solubility of the non‐interlocked oligo(*para‐*phenylene) derivatives. We assumed that the ring component of the rotaxane inhibited the aggregation, and the observed properties of the oligo(*para‐*phenylene) moiety of the [2]rotaxane would correspond to the properties of non‐aggregated oligo(*para‐*phenylene).

We envisioned that the [3]rotaxanes with two oligo(*para‐*phenylene) axles (type 2.1 [3]rotaxanes)[^21^, ^22^, ^23^, ^24^, ^25^, ^26^, ^27^, ^28^, ^29^, ^30^, ^31^, ^32^, ^33^, ^34^] could be suitable model compounds to understand the properties of aggregated oligo(*para‐*phenylene)s. In this study, we synthesized oligo(*para‐*phenylene)[3]rotaxanes by biaryl coupling mediated by the macrocyclic Ni complex (Scheme 1b). The use of a complex with a larger macrocycle was critical for the synthesis of [3]rotaxanes. The properties of [3]rotaxanes were analyzed by spectroscopic methods. The aggregation of the oligo(*para‐*phenylene) derivatives was examined.

## Results and Discussion

### Synthesis of Oligo(*para*‐phenylene)[3]rotaxanes

**Scheme 2:**
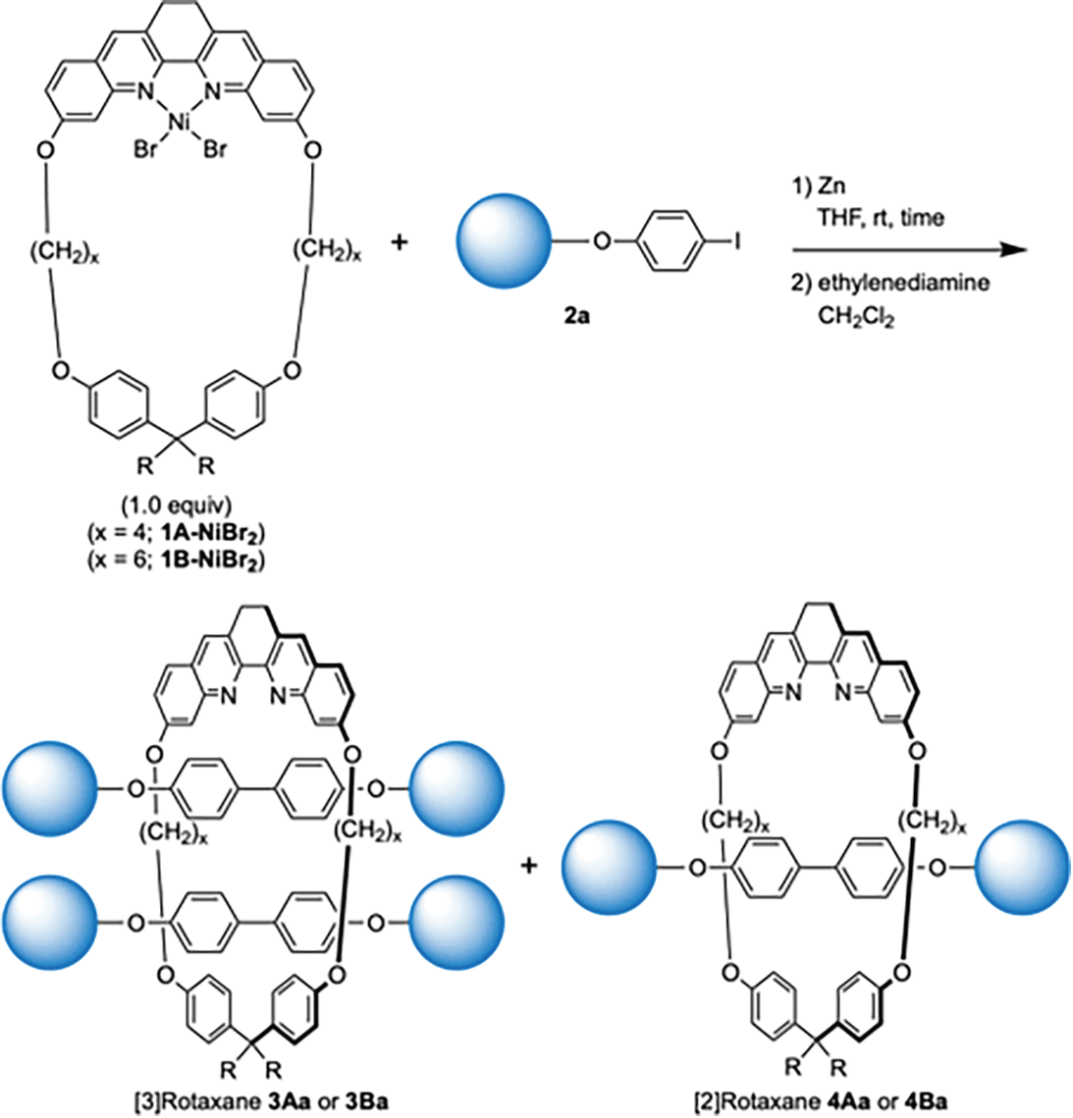
Optimization of the reaction conditions for the synthesis of [3]rotaxanes.

The study on the synthesis of biaryl[3]rotaxanes is summarized in Scheme 2. In our previous study, we synthesized [2]rotaxane **3Aa** by the reaction of macrocyclic dihydrodibenzophenanthroline Ni complex **1A‐NiBr_2_** (1.0 equiv.), iodoarene **2** **a** (2.5 equiv.), and Zn (3.0 equiv.) in THF at rt under Ar (entry 1). Though we anticipated that [3]rotaxane could be synthesized from **1A‐NiBr_2_** by using larger amounts of **2** **a** and Zn (6.0 equiv., each), we could not isolate [3]rotaxane **3Aa** and [2]rotaxane **4Aa** was isolated in 73 % yield (entry 2). Expecting that [3]rotaxane would be synthesized if we use a Ni complex with a larger macrocycle, we synthesized **1B‐NiBr_2_**, a macrocycle with a 35‐membered ring, and examined the synthesis of rotaxanes. When 2.5 equiv. of **2** **a** and 3.0 equiv. of Zn were used, [3]rotaxane **3Ba** was isolated in 6 % yield, and [2]rotaxane **4Ba** was the major product (33 % yield, entry 3). In this reaction, 26 % of macrocycle **1B** was recovered. The decreased combined yield of the rotaxane could be explained in terms of the flexible nature of the macrocycle: the increased flexibility of the ring component might prevent the efficient formation of rotaxane because the coupling reaction may not proceed in the cavity formed by the macrocycle.[^26^, ^35^] When the amounts of **2** **a** and Zn were increased to 6.0 equiv., **3Ba** was obtained in 57 % yield (entry 4). The yield of **3Ba** reached 74 % by increasing the amounts of **2** **a** and Zn to 8.0 equiv. (entry 5). It is noteworthy that the yield of the [3]rotaxane was higher compared to the yields of [3]rotaxanes reported by other methods.[^21^, ^22^, ^23^, ^24^, ^25^, ^26^, ^27^, ^28^, ^29^, ^30^, ^31^, ^32^, ^33^, ^34^, ^36^] The mild reaction conditions might have a favorable effect on the formation of [3]rotaxane. To reduce the amount of the iodoarene required for the reaction, we used 6.0 equiv. of the iodoarene (entry 4) for the synthesis of [3]rotaxanes in further studies.

**Scheme 3:**
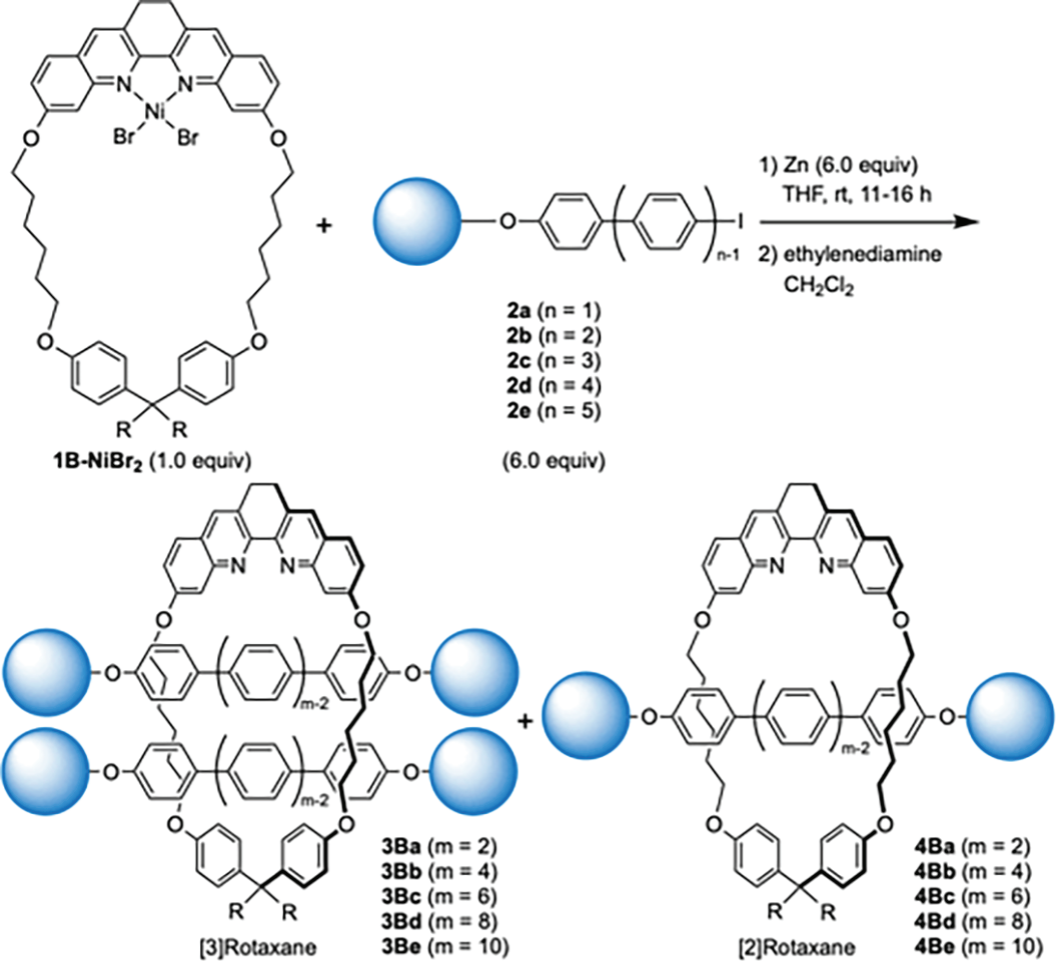
Synthesis of [3]rotaxanes with longer oligo(*para‐*phenylene) structures.

Next, the synthesis of [3]rotaxanes containing longer oligo(*para‐*phenylene) axle was examined (Scheme 3). When the axle precursor **2** **b** with PP2 structure was used, [3]rotaxane **3Bb** with PP4 axle was obtained in 54 % yield, and [2]rotaxane **4Bb** was obtained in 18 % yield (entry 2). Similarly, [3]rotaxane **3Bc** with PP6 structure was obtained in 49 % yield, and **4Bc** was obtained in 17 % yield when the axle precursor **2** **c** was used (entry 3). [3]Rotaxane with PP8 axle was synthesized in 41 % yield, and [2]rotaxane **4Bd** was not isolated (entry 4).^[37]^ When PP5 derivative **2** **e** was used, however, the formation of [3]rotaxane **3Be** was not observed, and [2]rotaxane **4Be** was isolated in 62 % yield.^[38]^

**Scheme 4:**
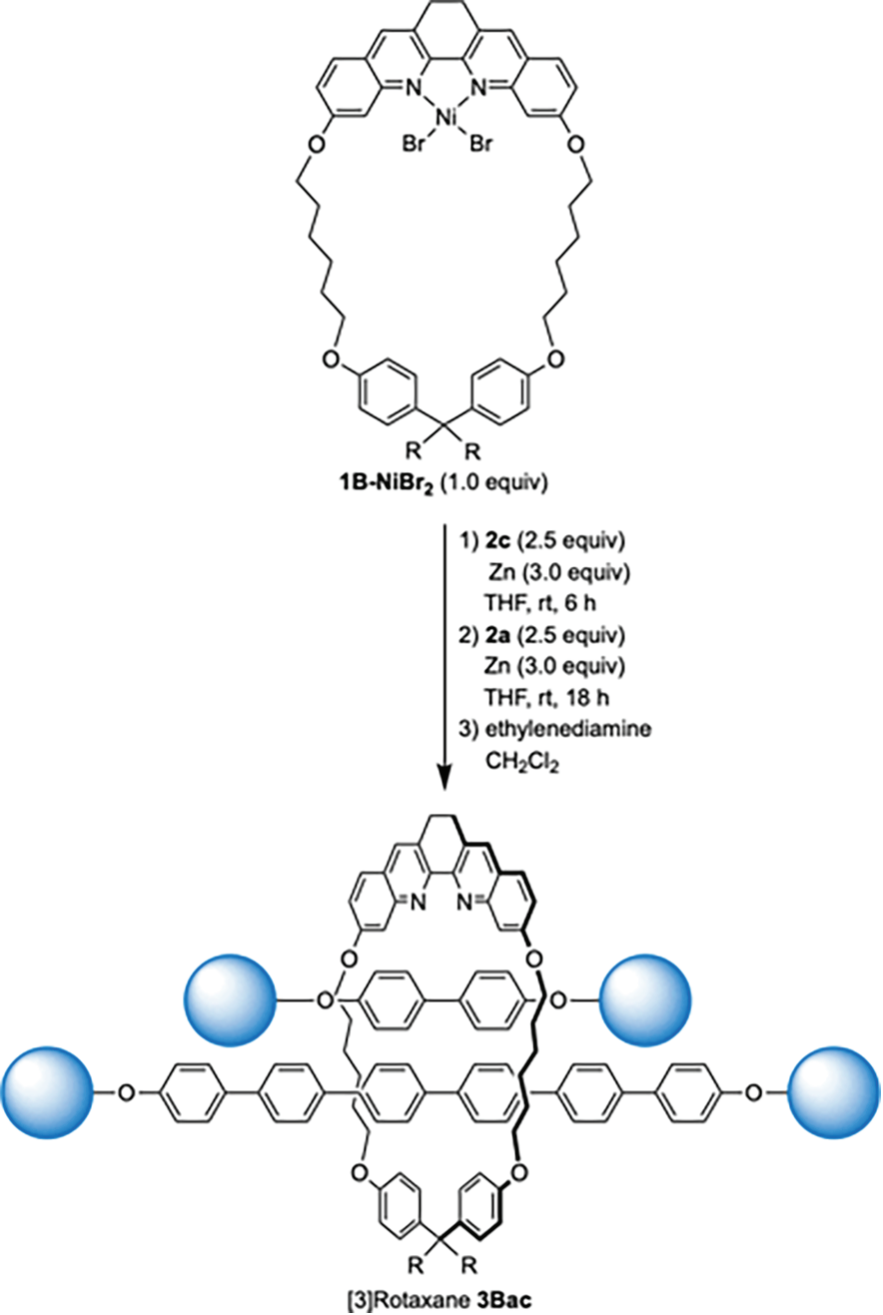
Synthesis of an oligo(*para‐*phenylene)[3]rotaxane with two different axles.

We applied this methodology to the synthesis of a [3]rotaxane with different phenylene axles (Scheme 4). The [2]rotaxane was synthesized *in situ* by the reaction of **1B‐NiBr_2_** (1.0 equiv.), PP3 axle precursor **2** **c** (2.5 equiv.), and Zn (3.0 equiv.). After the completion of the reaction, **2** **a** (2.5 equiv.) and Zn (3.0 equiv.) were added to the reaction mixture. The oligo(*para‐*phenylene) [3]rotaxane **3Bac** with two different axles was isolated in 21 % yield.

### ^1^H NMR Analysis

**Figure 1:**
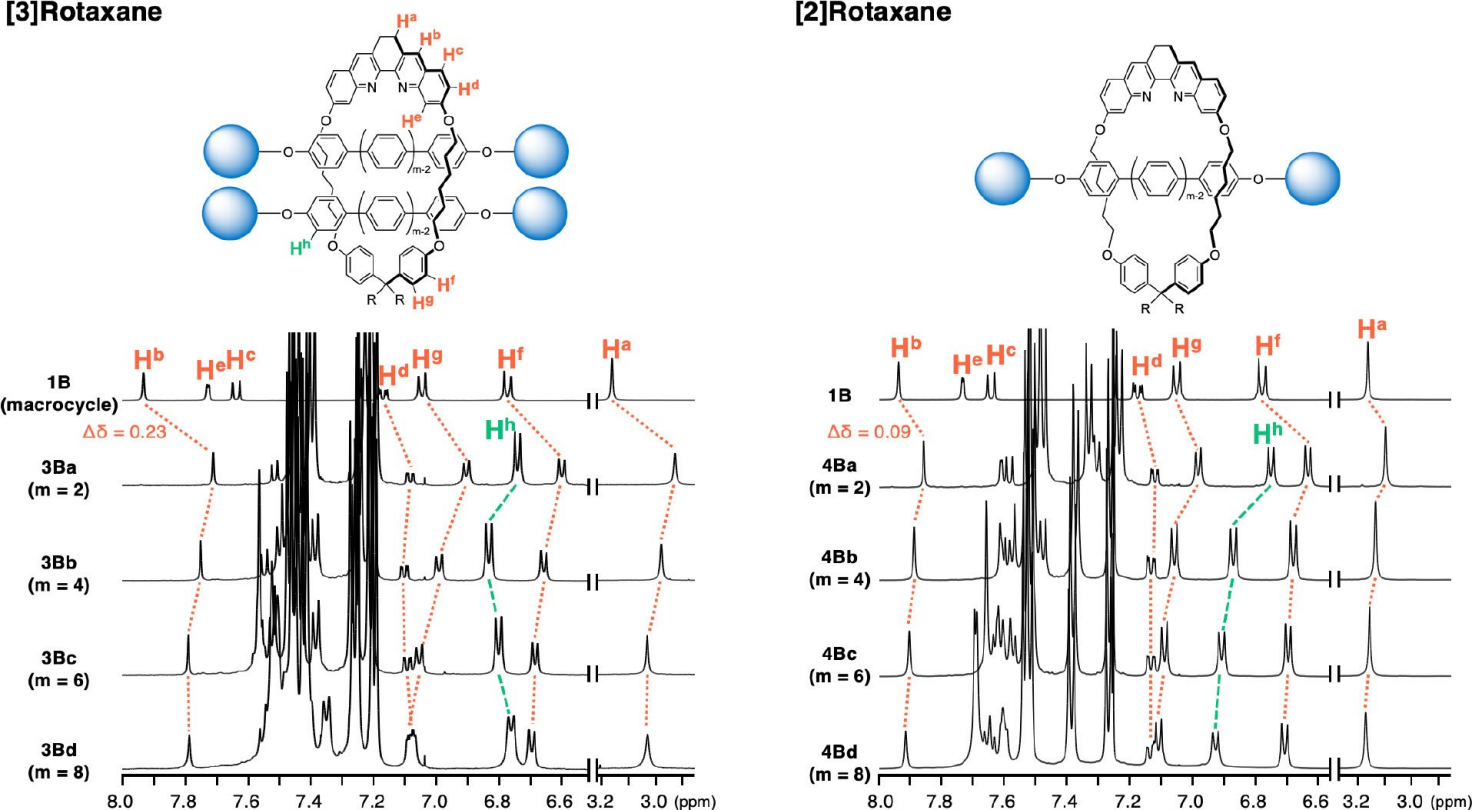
Comparison of ^1^H NMR spectra of macrocycle **1B**, [3]rotaxane **3Ba‐d** and [2]rotaxane **4Ba‐d** (500 MHz, CDCl_3_, 24 °C).

We compared the ^1^H NMR spectra of [3]rotaxanes **3Ba**‐**d** and [2]rotaxanes **4Ba**‐**d**, and the results are summarized in Figure 1. The signals of macrocycle **1B** shifted upfield in the rotaxanes. For example, the signal of the proton assigned to H^b^ of **1B** shifted to higher field in [3]rotaxane **3Ba** (ΔH^b^ (**1B**‐**3Ba**)=0.23 ppm).^[39]^ Compared to the difference of H^b^ in **1B** and [2]rotaxane **4Ba** (ΔH^b^ (**1B**‐**4Ba**)=0.09 ppm), the difference was more pronounced. The result could be explained in terms of the presence of the dumbbell moiety (two triarylmethyl groups) of the axle in the proximity of the ring component in the rotaxanes. The dumbbell moiety would induce high‐field shift of the signals assigned to the protons bound to the ring component. The influence is larger in [3]rotaxane **3Ba** since two axles are present in **3Ba**. In a rotaxane with a longer axle component, the influence of the dumbbell moiety would be diminished, and this phenomenon was observed in the NMR spectra of longer rotaxanes. Compared to the chemical shift of H^b^ in short rotaxanes (**3Ba** and **4Ba**), the low‐field shift of H^b^ was observed in longer [3]rotaxanes and [2]rotaxanes. Similar shift was observed in other signals assigned to protons bound to the macrocycle.^[40]^ We next examined the difference of the chemical shifts of signals assigned to the oligo(*para‐*phenylene) moiety. Though most of the signals assigned to the oligo(*para‐*phenylene) moiety of the rotaxanes were overlapped, the signals of the proton assigned to H^h^ of the axle did not overlap with other signals. As shown in Figure 1, the relationship between the chemical shift of H^h^ and the structure of the rotaxanes is influenced by the number of the threaded axles as well as the length of the oligo(*para*‐phenylene) moiety.

**Figure 2:**
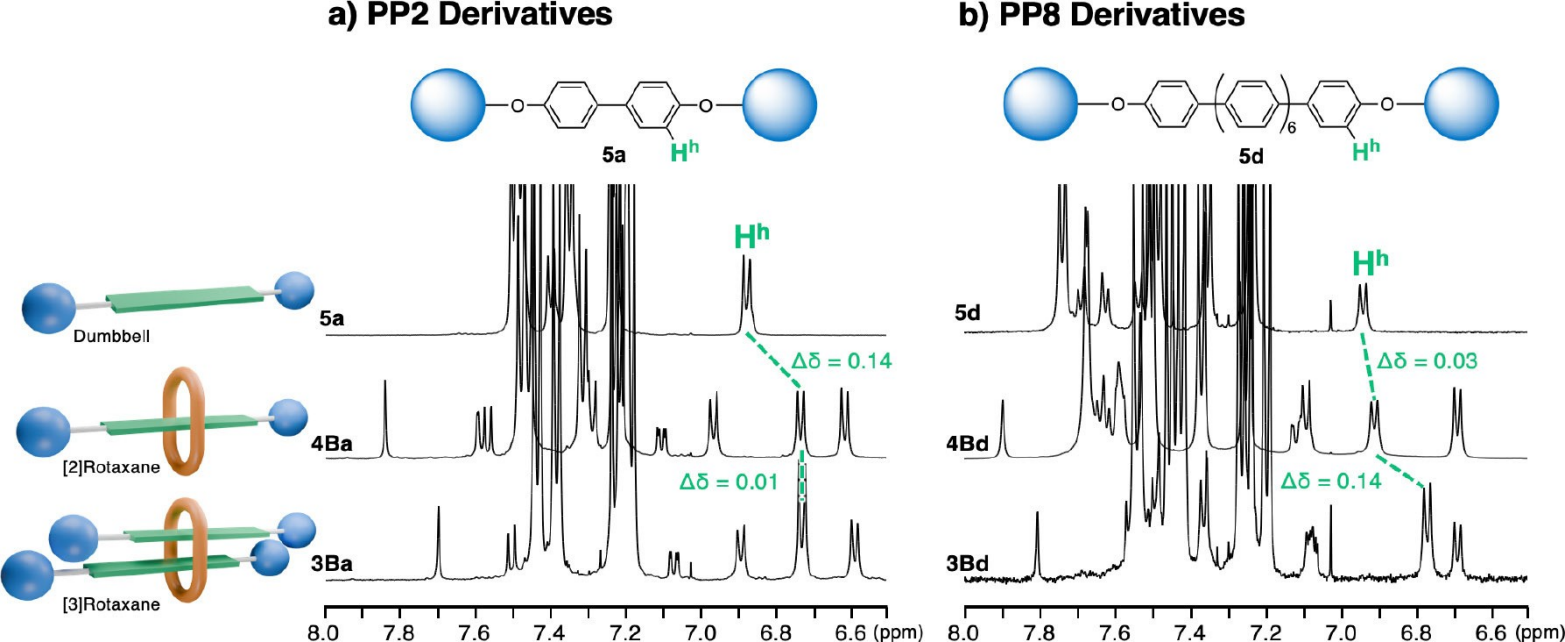
Comparison of ^1^H NMR of PP2 derivatives (**5** **a**, **4Ba** and **3Ba**), and PP8 derivatives (**5** **d**, **4Bd** and **3Bd**).

To discuss the result in detail, we compared the chemical shift of compounds which have same oligo(*para‐*phenylene) structure (Figure 2). In Figure 2a, we compared the dumbbell compound with PP2 structure (**5** **a**), [2]rotaxane with PP2 axle (**4Ba**), and [3]rotaxane with PP2 axle (**3Ba**). The signal assigned to H^h^ in **5** **a** shifted upfield in **4Ba**, and the presence of the macrocycle would have induced this high‐field shift. The difference of the chemical shift of H^h^ in **4Ba** and **3Ba** is negligible, implying that the influence of another dumbbell moiety on the chemical shift of H^h^ is small. We assume that the interaction of the biphenylene groups is weak in [3]rotaxane **3Ba**. In Figure 2b, we examined the NMR spectra of the dumbbell compound with PP8 structure (**5** **d**), [2]rotaxane with PP8 axle (**4Bd**), and [3] rotaxane with PP8 axle (**3Bd**). The signal assigned to H^h^ in **5** **d** was not affected significantly when the ring component was introduced (ΔH^b^ (**5** **d**‐**4Bd**)=0.03 ppm). This result could be explained by the reduced influence of the macrocycle on the axle component in a longer rotaxane: the probability of the presence of the macrocycle in the proximity of H^h^ would be reduced in a longer rotaxane. The difference of the chemical shift of H^h^ in **4Bd** and **3Bd** is large (ΔH^b^ (**4Bd**‐**3Bd**)=0.14 ppm), and this result contrasts with the results reported for PP2 derivatives (Figure 2a). We assume that the interaction of the octaparaphenylene groups is strong in [3]rotaxane **3Bd**, inducing the high‐field shift of the signal assigned to H^h^.^[41]^

### Aggregation Behavior

**Figure 3:**
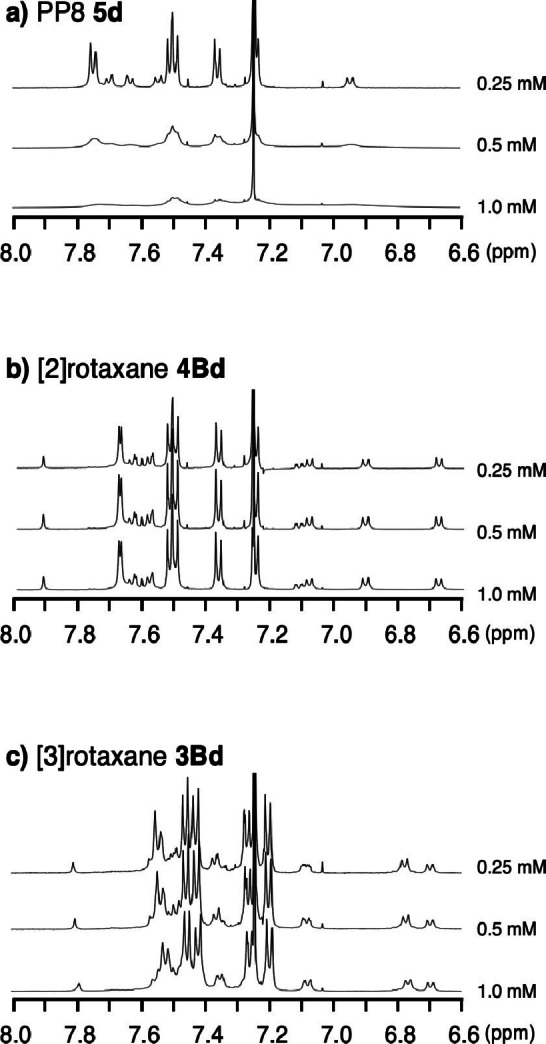
^1^H NMR spectra of **5** **d**, **4Bd** and **3Bd** at 0.25 mM, 0.50 mM, and 1.0 mM in CDCl_3_ at 24 °C.

Longer oligo(*para‐*phenylene) tends to aggregate and has low solubility due to the π‐π stacking interaction between phenylene moieties. The presence of the macrocycle in the rotaxanes may inhibit the intermolecular π‐π interaction. To study the aggregation behavior of the oligo(*para*‐phenylene) derivatives in detail, we examined the influence of the concentration of octa(*para*‐phenylene) **5** **d**, [2]rotaxane **4Bd**, and [3]rotaxane **3Bd** on the ^1^H NMR spectra, and the results are summarized in Figure 3. A sharp spectrum of **5** **d** was observed in CDCl_3_ at low concentration (0.25 mM), while the broadening of the signals was observed when the NMR spectrum was observed at higher concentration (0.50 mM or 1.0 mM, Figure 3a). In contrast, the broadening of the signals was not observed when the spectra of **4Bd** and **3Bd** were recorded at high concentration. The results could be explained in terms of the aggregation of **5** **d** at high concentration.

**Figure 4:**
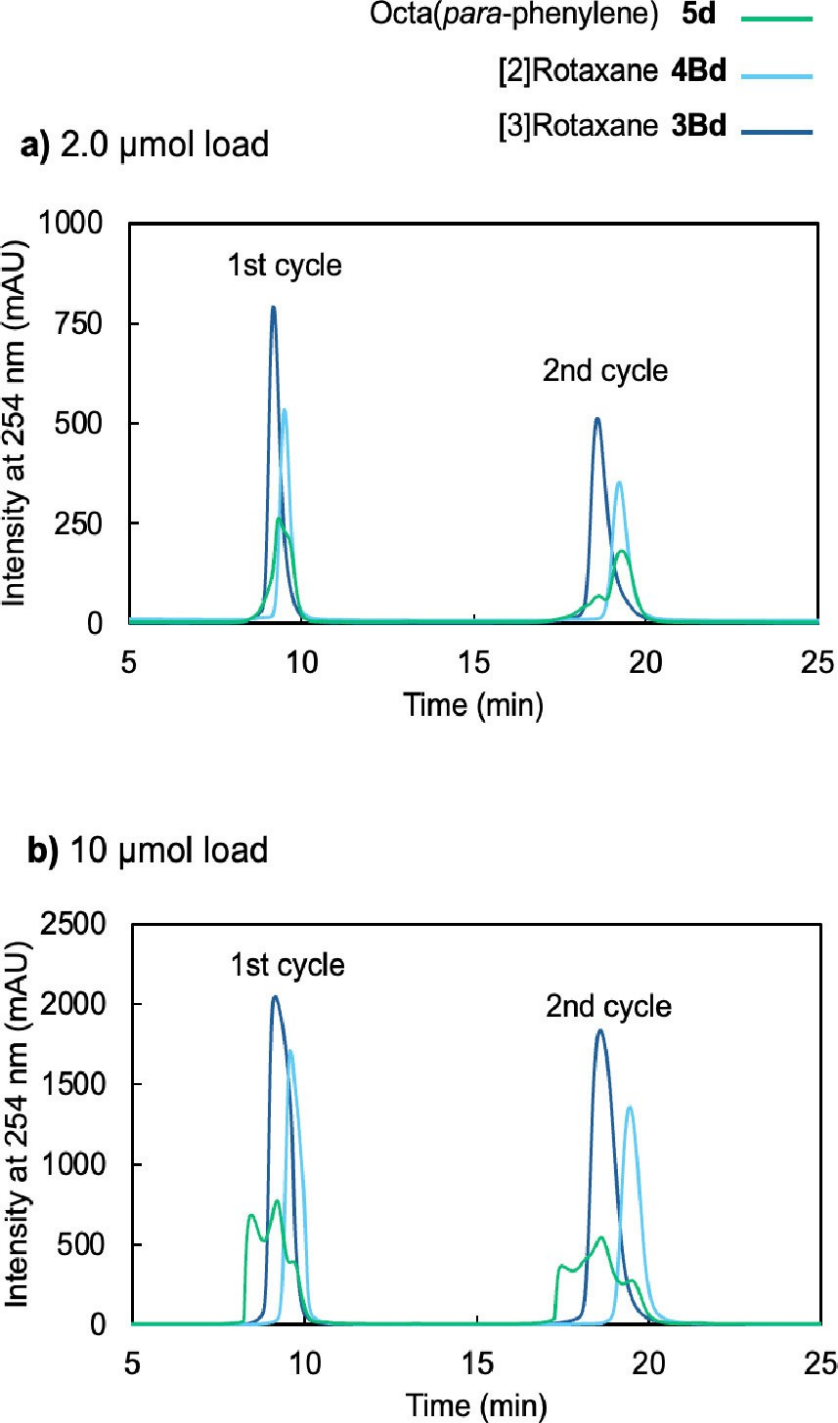
Recycle GPC trace of **5** **d**, **4Bd** and **3Bd** with different loads.

The aggregation of **5** **d** was also suspected in the recycle gel permeation chromatography (GPC) trace (Figure 4). The retention time of **5** **d** was comparable to those of **4Bd** or **3Bd** when a smaller amount (2.0 μmol) of **5** **d** was loaded (Figure 4a). When a larger amount (10 μmol) of **5** **d** was loaded, the retention time of **5** **d** was shorter than those of **4Bd** or **3Bd**, implying the formation of the aggregate (Figure 4b).

**Figure 5:**
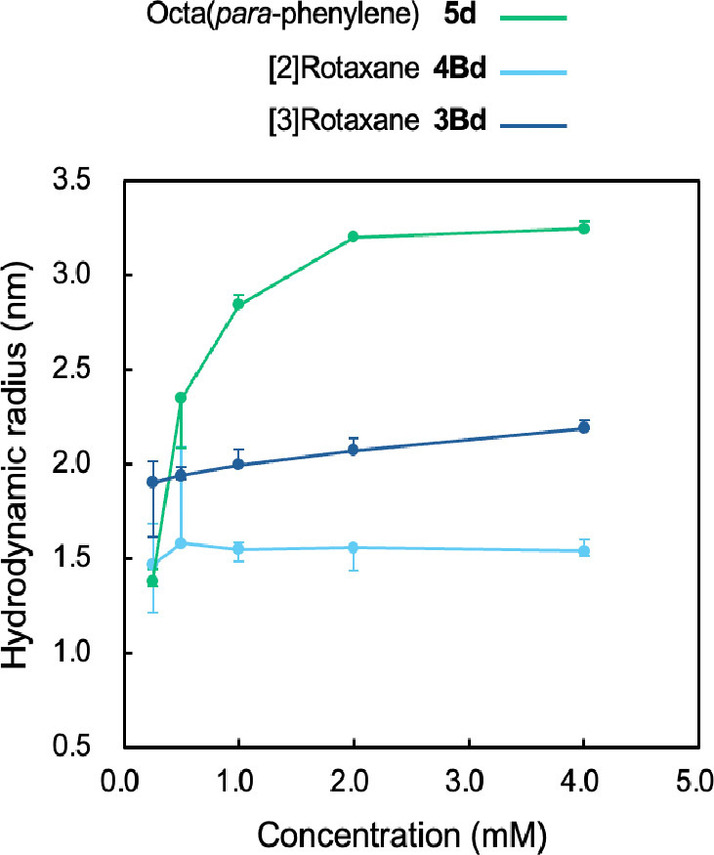
Hydrodynamic radius (nm) of PP8 derivatives **5** **d**, **4Bd**, and **3Bd** measured by DLS at 25 °C in CHCl_3_.

To study the aggregation of the compounds in detail, we measured the hydrodynamic radius (Rh) of **5** **d**, **4Bd** and **3Bd** by DLS (Dynamic Light Scattering) at 25 °C in chloroform. Measurements were conducted at four different concentrations (0.25, 0.50, 1.0, 2.0 and 4.0 mM), and the results are summarized in Figure 5. The Rh of PP8 **5** **d** showed significant concentration dependence. At 0.25 mM, the Rh of **5** **d** reached a value of 1.4 nm, comparable to the value (1.5–1.6 nm) of [2]rotaxane **4Bd**. From 0.25 mM to 1.0 mM, the Rh of **5** **d** increased, and at 2.0 and 4.0 mM, **5** **d** had a larger Rh of 3.2 nm compared to the other molecules. The result implies that the aggregation of **5** **d** would occur at high concentration (0.50–4.0 mM). In contrast, the Rh of [2]rotaxane **4Bd** and [3]rotaxane **3Bd** remained almost constant, implying that the aggregation of the compounds would not occur even at high concentration (4.0 mM). The Rh of [2]rotaxane **4Bd** was 1.5‐1.6 nm, which is considered to reflect the size of a single **4Bd** molecule. The Rh of [3]rotaxane **3Bd** was 1.9–2.2 nm, reflecting the larger size of the molecule compared to **4Bd**.^[42]^ Based on these results, we assume that the intermolecular π‐π interaction between the octa(*para*‐phenylene) moieties was suppressed by the presence of the ring component in rotaxanes **4Bd** and **3Bd**.

### Photophysical Properties

**Figure 6:**
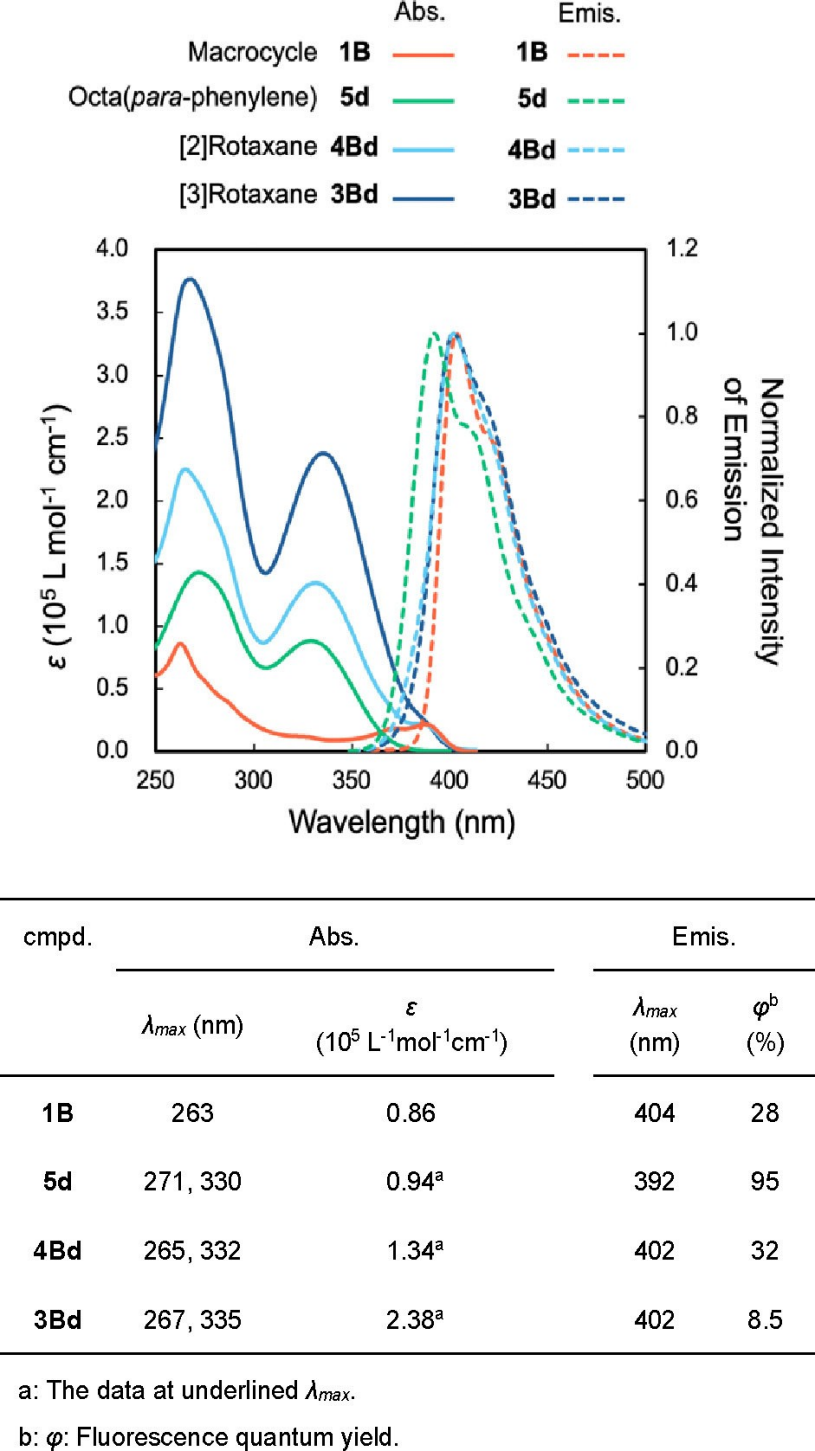
Photophysical properties of PP8 derivatives (5.0 μM in CHCl_3_).

The photophysical properties of macrocycle **1B** and PP8 derivatives (dumbbell compound **5** **d**, [2]rotaxane **4Bd**, and [3]rotaxane **3Bd**) were examined and the results are summarized in Figure 6. In the absorption spectrum of **5** **d**, an absorption assigned to the dumbbell moiety (4‐cyclohexylbiphenyl moiety) at 271 nm and an absorption assigned to the octa(*para*‐phenylene) moiety[^8^, ^11^] at 330 nm were observed. The absorption spectrum of [2]rotaxane **4Bd** was similar to the sum of the spectra of **1B** and **5** **d**. Similarly, the spectrum of [3]rotaxane **3Bd** resembled the sum of the spectra of **1B** and “doubled” **5** **d**. The results imply that the influence of the threaded structure or the presence of the octa(*para*‐phenylene) moieties in the proximity is very small.

In the emission spectra of **1B**, the emission delivered from the dihydrodibenzophenanthroline structure was observed at 404 nm. In the emission spectrum of **5** **d**, the PP8‐derived emission was observed at 392 nm.[^8^, ^11^] In [3]rotaxane **3Bd**, the emission derived from PP8 overlapped with the emission derived from the dihydrodibenzophenanthroline structure, which was observed at 402 nm. Similarly, the overlapped emission was observed at 402 nm in **4Bd**. Based on these results, we concluded that the difference of the *λ_max_* of the compounds which have PP8 moiety (**5** **d**, **4Bd** and **3Bd**) is small. In contrast, the quantum yields of **5** **d**, **4Bd** and **3Bd** were quite different. The fluorescence quantum yield of macrocycle **1B** was 28 %, and the fluorescence quantum yield of octaparaphenylene **5** **d** was 95 %. The observed high value for **5** **d** is comparable to the reported values for long oligo(*para‐*phenylene)s.^[7]^ The fluorescence quantum yield of **4Bd** decreased to 32 %, although it has the PP8 moiety. This result implies that in [2]rotaxane **4Bd**, quenching might have occurred by the presence of the axle and ring components in proximity. In [3]rotaxane **3Bd**, the fluorescence quantum yield was very low (8.5 %). We assume that the strong quenching occurred because of the presence of two phenylene moieties and the ring component in the proximity. The observed phenomena should be related to aggregation‐caused quenching (ACQ), which is frequently observed for π‐conjugated luminescent compounds. It has been assumed that ACQ was induced by the excited state decay in the aggregate to the ground state via non‐radiative pathway.[^43^, ^44^] The results imply that strong quenching would be observed when the emission spectrum of an oligo(*para‐*phenylene) derivative was recorded in aggregated or solid state.

## Conclusions

We synthesized a series of [3]‐ and [2]rotaxanes with various length of oligo(*para*‐phenylene) axle by the biaryl coupling reaction of iodoarenes mediated by a macrocyclic dihydrodibenzophenanthroline‐Ni complex. The structure of the rotaxanes was examined in detail by ^1^H NMR studies. We disclosed that aggregation of the oligo(*para*‐phenylene) derivative, which was frequently reported in the chemistry of long oligo(*para*‐phenylene)s, was suppressed in the rotaxanes. The strong quenching of the emission was observed in the [3]rotaxane with two oligo(*para*‐phenylene) axles, which would reflect the property of the aggregated structure. By utilizing the interlocked structure, it is now possible to analyze the properties of the aggregated PPn in a dilute solution and study the properties of non‐aggregated PPn at high concentration: the aggregation/non‐aggregation state could be controlled. The study would contribute to the understanding of the properties of oligo(*para*‐phenylene)s. Further studies related to the synthesis and properties of interlocked π‐conjugated compounds are in progress.

## Supporting Information

Experimental section; characterization data for all new compounds; ^1^H NMR and ^13^C NMR spectra. Details of GPG and DLS analysis. Auto correlation function of DLS analysis. Result of SLS analysis (PDF).

## Conflict of Interests

The authors declare no conflict of interest.

1

## Supporting information

As a service to our authors and readers, this journal provides supporting information supplied by the authors. Such materials are peer reviewed and may be re‐organized for online delivery, but are not copy‐edited or typeset. Technical support issues arising from supporting information (other than missing files) should be addressed to the authors.

Supporting Information

## Data Availability

The data that support the findings of this study are available from the corresponding author upon reasonable request.
