# Supplementary material for: Synthesis and Properties of [3]Rotaxanes with Two Oligo(para‐phenylene) Axles
Source: Chemistry. 2025 Apr 14;31(25):e202500522. doi: 10.1002/chem.202500522 (PMC12057601; doi:10.1002/chem.202500522)
Supplement: Supplementary file 1 — Supporting Information [file CHEM-31-e202500522-s001.pdf]

Chemistry–A European Journal

Supporting Information

Synthesis and Properties of [3]Rotaxanes with Two Oligo(*para*-phenylene) Axles

Misuzu Ohta, Ayano Okuda, Kayori Takahashi, Shoichi Hosoya, Yusuke Yoshigoe, and Shinichi Saito*

Supporting Information

Synthesis and Properties of [3]Rotaxanes with Two Oligo(*para*-phenylene) Axles

Misuzu Ohta,[a] Ayano Okuda,[a] Kayori Takahashi,[b] Shoichi Hosoya,[c] Yusuke Yoshigoe,[a] and Shinichi Saito*,[a]

[a] Department of Chemistry, Faculty of Science, Tokyo University of Science, 1-3 Kagurazaka, Shinjuku, Tokyo 162-8601, Japan

[b] National Metrology Institute of Japan (NMIJ), National Institute of Advanced Industrial Science and Technology (AIST), Tsukuba Central 3, 1-1-1 Umezono, Tsukuba, Ibaraki 305-8563, Japan

[c] Ochanomizu Research Facility, Bioscience Center, Research Infrastructure Management Center, Institute of Science Tokyo, 1-5-45 Yushima, Bunkyo-ku, Tokyo 113-8510, Japan

*Email: ssaito@rs.tus.ac.jp

Contents

1. General Information S2

2. Experimental Section
    2.1 Synthesis of Macrocyclic $NiBr_2$ complex **1B-NiBr₂** S3
    2.2 Synthesis of [3]- and [2]rotaxanes S6

3. ^{1}H NMR and ^{13}C NMR spectra S18

4. Details of GPC analysis S30

5. DLS and SLS
    5.1 Details of DLS analysis S31
    5.2 Autocorrelation function S32
    5.3 SLS analysis of **5d**, **4Bd**, and **3Bd** S35

1. General Information

Moisture and air-sensitive reactions were performed using standard syringe-septum or schlenk technique under Ar atmosphere unless otherwise noted. Oil bath was used as the heating source and the external temperature was reported. Commercially available reagents were used without further purification unless otherwise noted. Zn powder was washed with HCl aq. and Et_2O, dried under vaccum, and stored under Ar. All reactions were monitored by thin-layer chromatography (TLC, on Merck silica gel 60F-254 plates) and visualization of the spots was done under UV light or by dipping the plates in polymolybidic acid- ethanol or anisaldehyde-acid reagent and heating at ~120 °C. Column chromatography was performed using silica gel 60N (spherical, neutral 40−50 μm) or silica gel 60 (spherical, acidic 40−50 μm) from Kanto Chemicals. NMR spectra were recorded on a JEOL 300, 400 or 500 MHz spectrometer or a Bruker 400 MHz NMR spectrometer. Chemical shifts were reported in delta units (δ) relative to chloroform (7.24 ppm for ^{1}H NMR and 77.23 ppm for ^{13}C NMR) or dimethyl sulfoxide (DMSO, 2.50 ppm for ^{1}H NMR and 39.5 ppm for ^{13}C NMR) as internal reference standards. Multiplicity is indicated by s (singlet), d (doublet), t (triplet), q (quartet), quint (quintet), m (multiplet), or bs (broad singlet). Coupling constants (J) are reported in Hertz. IR spectra were recorded on a Fourier transform infrared spectrometer using a diamond ATR module. A YMC-GPC T30000 (21.2 mm ID × 600 mm L) column was used for GPC separation using $CHCl_3$ as the eluent. High-resolution mass spectra (HRMS) were obtained by using matrix-assisted laser desorption/ionization (MALDI), Electrospray ionization (ESI), Electron ionization (EI) and a time-of-flight (TOF) mass analyzer. UV-vis spectra were recorded on a UV-3150PC (SHIMAZU) at rt. Emission spectra were recorded on a RF-6000 (SHIMAZU) at rt. Fluorescence quantum yields were recorded on a C-9920-02G (Hamamatsu photonics). Dynamic-light-scattering (DLS) and static-light-scattering (SLS) were recorded on a ALV/DLS/SLS-5000F-SO-SIPD (ALV-LaserVertriebsgesellschaft m.b.H). For synthesis of compounds **1A-NiBr** and **2a**-**e**, see, ref. 11.

2. Experimental Section

2.1 Synthesis of Macrocyclic $NiBr_2$ complex **1B-NiBr₂**

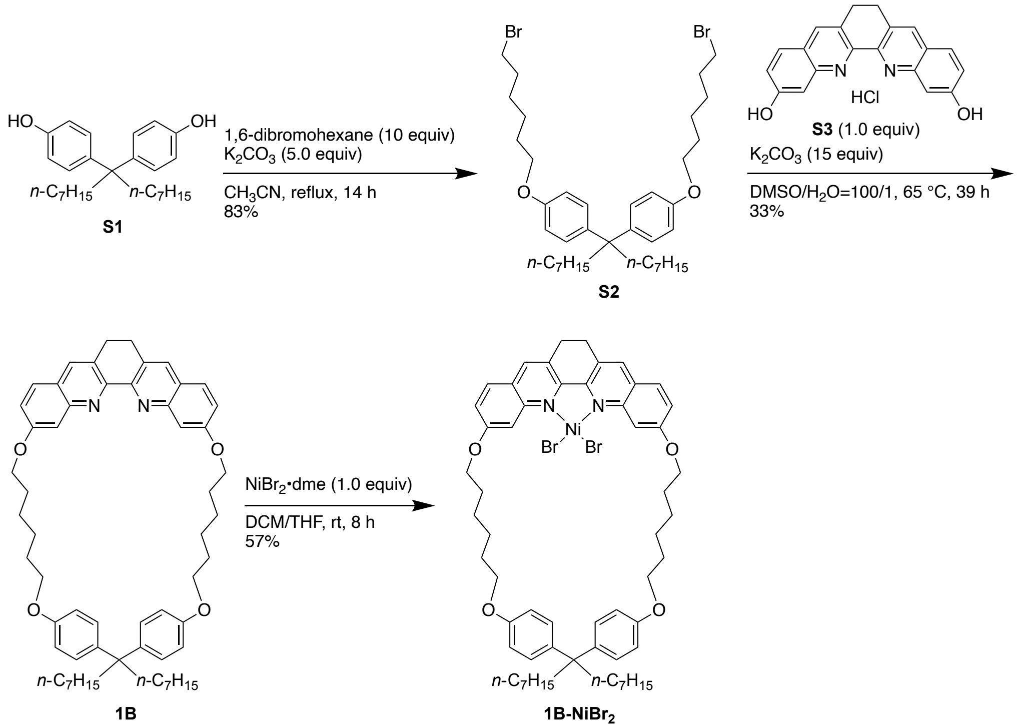

Scheme S1. Synthesis of macrocyclic complex **1B-NiBr₂**

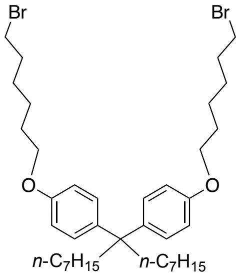

S2

S2

This experiment was performed under air. 4,4'-(Pentadecane-8,8-diyl)diphenol (1.00 g, 2.5 mmol), 1,6-dibromohexane (3.9 mL, 25 mmol), and K_2CO_3 (1.70 g, 13 mmol) were dissolved in CH_3CN (70 mL). The reaction mixture was refluxed for 14 h. The mixture was filtered through celite, and the filtrate was concentrated *in vacuo*. The crude mixture was purified by flash column chromatography over silica gel (hexane, then hexane : CH_2Cl_2 = 3 : 1) to give a colorless oil. Yield: 1.4 g (2.1 mmol, 83%).

^{1}H NMR (400 MHz, $CDCl_3$): δ 7.04 (d, J = 8.7 Hz, 4H), 6.75 (d, J = 8.7 Hz, 4H), 3.91 (t, J = 6.4 Hz, 4H), 3.40 (t, J = 6.8 Hz, 4H), 1.98-1.84 (m, 8H), 1.78-1.75 (m, 4H), 1.50-1.48 (m, 8H), 1.26-1.18 (m, 16H), 0.93-0.93 (m, 4H), 0.84 (t, J = 7.1 Hz, 6H).

^{13}C NMR (100 MHz, $CDCl_3$): δ 156.8, 141.7, 129.0, 113.6, 67.7, 48.2, 38.1, 34.0, 32.9, 32.1, 30.6, 29.4, 28.2, 25.6, 24.1, 22.8, 14.3.

IR (ATR): 2932, 2857, 1608, 1510 cm^{-1}.

HRMS (ESI-TOF) calcd. for $C_{39}H_{66}{}^{79}Br_2NO_2$ ($[M+NH_4]^+$): 738.3455, found 738.3468.

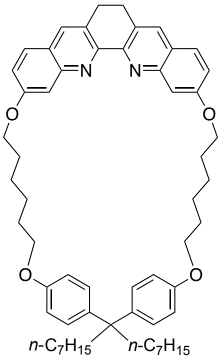

1B

1B

This experiment was performed under air. To a mixture of **S2** (355 mg, 0.49 mmol) and **S3** (172.2 mg, 0.49 mmol) in DMSO (100 mL) and H_2O (1.0 mL) was added K_2CO_3 (1.018 mg, 7.4 mmol). The mixture was stirred at 65 °C for 39 h. The solvent was removed under reduced pressure, and the residue was extracted with CH_2Cl_2. The organic

layer was washed with H_2O and brine, dried over Na_2SO_4 and concentrated *in vacuo*. The crude mixture was purified by flash column chromatography over acidic silica gel (hexane : MeOH = 60 : 1) to give a yellow solid. Yield: 163 mg (0.19 mmol, 33%).

mp: 79.6-80.6 °C

^{1}H NMR (400 MHz, $CDCl_3$): δ 7.93 (s, 2H), 7.73 (d, J = 2.3 Hz, 2H), 7.64 (d, J = 9.1 Hz, 2H), 7.17 (dd, J = 9.1, 2.3 Hz, 2H), 7.04 (d, J = 8.7 Hz, 4H), 6.77 (d, J = 9.1 Hz, 4H), 4.09 (t, J = 6.8 Hz, 4H), 3.95 (t, J = 6.4 Hz, 4H), 3.16 (s, 4H), 2.01-1.79 (m, 12H), 1.53-1.52 (m, 8H), 1.26-1.17 (m, 16H), 0.97-0.95 (m, 4H), 0.86-0.80 (m, 6H).

^{13}C NMR (100 MHz, $CDCl_3$): δ 159.8, 156.6, 152.7, 150.0, 142.1, 134.5, 130.5, 128.8, 127.8, 123.8, 121.1, 113.9, 109.6, 68.2, 67.5, 47.9, 37.4, 32.1, 30.6, 29.4, 29.3, 29.1, 28.7, 25.6, 25.4, 24.1, 22.8, 14.3.

IR (ATR): 2930, 2857, 1621, 1507 cm^{-1}.

HRMS (ESI-TOF) calcd. for $C_{59}H_{75}N_2O_4$ ([M+H]$^+$): 875.5721, found 875.5719.

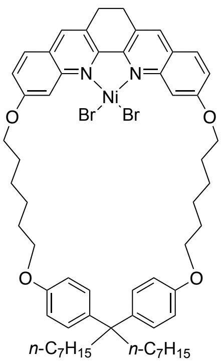

1B-NiBr₂

Macrocyclic complex 1B-NiBr$_2$

To a solution of **1B** (163 mg, 0.19 mmol) in dry CH_2Cl_2 (1 mL) was added a solution of $NiBr_2$•dme (57.6 mg, 0.19 mmol) in dry THF (3 mL) at rt under Ar. The mixture was stirred at rt for 8 h, and the precipitate was collected by filtration. The solid was washed with CH_3OH and H_2O to give an orange powder. Yield: 116.8 mg (0.11 mmol, 57%)

mp: 239.7 °C (decomp)

IR (ATR): 2933, 2858, 1621, 1505, 1234 cm^{-1}.

Anal. calcd. for $C_{59}H_{74}Br_2N_2NiO_4$: C, 64.79; H, 6.82; N, 2.56. found: C, 64.67; H, 6.90; N, 2.57.

2.2 Synthesis of [3]- and [2]rotaxanes

General procedure for the synthesis of [3]- and [2]rotaxanes

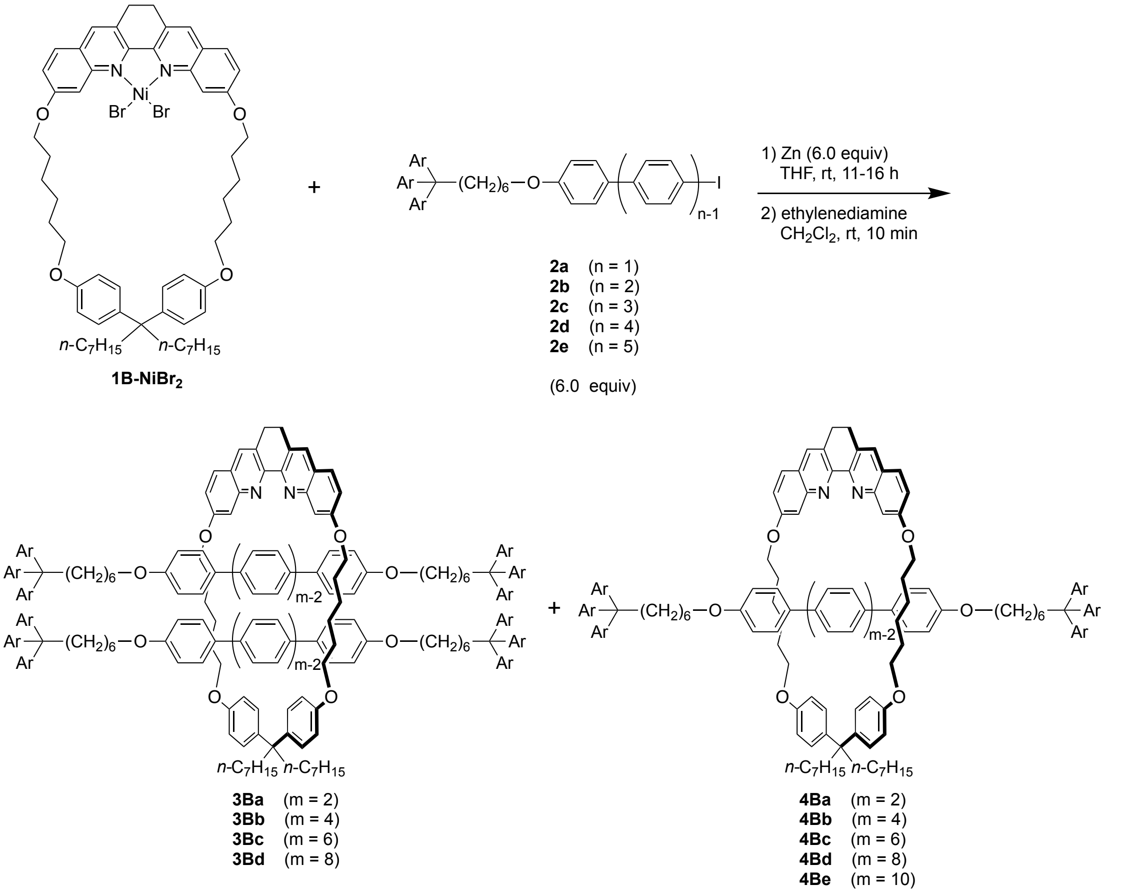

To a suspension of **1B-NiBr$_2$** (11 mg, 10 μmol) in dry THF (0.5 mL) was added Zn powder (3.9 mg, 60 μmol) at rt under Ar, and the mixture was sonicated for 3 min. To the dark green mixture was added **2** (60 μmol) and the resulting mixture was stirred at rt for 11-16 h. To the mixture was added CH_2Cl_2 (2.0 mL) and ethylenediamine (2.0 mL), and the mixture was stirred at rt for 10 min. The resulting mixture was extracted with CH_2Cl_2. The organic layer was washed with brine, dried over anhydrous Na_2SO_4 and concentrated *in vacuo*. The crude mixture was purified by flash column chromatography over silica gel (hexane : CH_2Cl_2 = 3 : 1, then CH_2Cl_2 : CH_3OH = 20 : 1) and gel permeation chromatography.

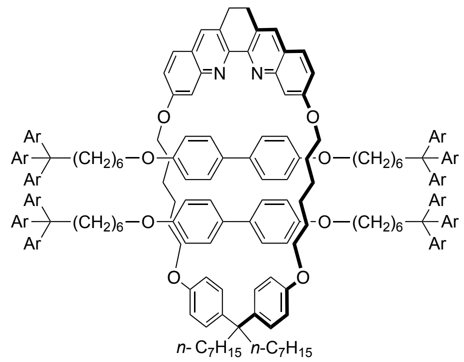

3Ba

3Ba

2a (61.3 mg, 60 μmol) was taken, and the mixture was stirred for 14 h to give a pale-yellow solid. Yield: 25.4 mg (5.7 μmol, 57%).

mp: 161.7-163.3 °C

^{1}H NMR (500 MHz, $CDCl_3$): δ 7.70 (s, 2H), 7.51 (d, *J* = 8.6 Hz, 2H), 7.45-7.43 (m, 32H), 7.40-7.38 (m, 26H), 7.20-7.18 (m, 48H), 7.07 (dd, *J* = 8.6, 2.3 Hz, 2H), 6.89 (d, *J* = 8.6 Hz, 4H), 6.73 (d, *J* = 8.6 Hz, 8H), 6.59 (d, *J* = 8.6 Hz, 4H), 3.81-3.75 (m, 8H), 3.63 (t, *J* = 6.6 Hz, 8H), 2.92 (s, 4H), 2.50-2.39 (m, 20H), 1.87-1.66 (m, 80H), 1.44-1.33 (m, 72H), 1.24 (m, 8H), 1.10 (brs, 16H), 0.97 (brs, 12H), 0.83-0.77 (m, 10H).

^{13}C NMR (125 MHz, $CDCl_3$): δ 159.9, 158.2, 156.7, 153.1, 150.2, 147.2, 146.5, 141.8, 138.5, 138.4, 133.9, 132.9, 130.5, 129.7, 128.5, 127.8, 127.5, 127.3, 127.0, 126.4, 123.6, 120.6, 114.9, 113.8, 109.2, 68.1, 67.1, 56.1, 47.3, 44.4, 40.5, 36.7, 34.7, 32.1, 30.6, 30.5, 29.7, 29.6, 29.5, 29.4, 28.7, 27.1, 26.4, 26.0, 25.6, 25.5, 23.9, 22.9, 14.3 (two signals are missing).

IR (ATR): 2924, 2850, 1497 cm^{-1}.

HRMS (MALDI-TOF) calcd. for $C_{327}H_{367}N_2O_8$ ($[M+H]^+$): 4449.8367, found 4449.8446.

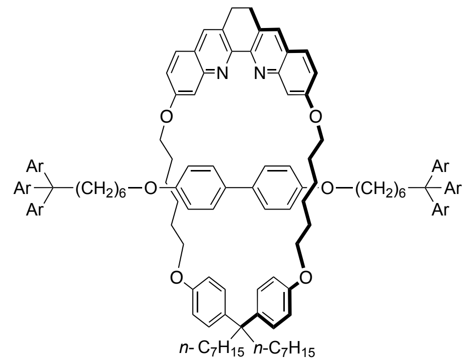

4Ba

4Ba

Pale-yellow solid. Yield: 4.8 mg (1.8 μmol, 18%).

mp: 119.2-119.9 °C

^{1}H NMR (500 MHz, $CDCl_3$): δ 7.84 (s, 2H), 7.60-7.56 (m, 4H), 7.49-7.45 (m, 24H), 7.33-7.28 (m, 16H), 7.22 (d, *J* = 8.9 Hz, 12H), 7.11 (dd, *J* = 9.2, 2.3 Hz, 2H), 6.97 (d, *J* = 8.6 Hz, 4H), 6.74 (d, *J* = 8.6 Hz, 4H), 6.62 (d, *J* = 8.6 Hz, 4H), 3.88 (t, *J* = 6.9 Hz, 4H), 3.74 (t, *J* = 6.6 Hz, 4H), 3.68 (t, *J* = 6.7 Hz, 4H), 3.09 (s, 4H), 2.56-2.48 (m, 10H), 1.96-1.82 (m, 28H), 1.75-1.55 (m, 26H), 1.46-1.14 (m, 58H), 0.91 (brs, 4H), 0.80 (t, *J* = 7.2 Hz, 6H).

^{13}C NMR (125 MHz, $CDCl_3$): δ 159.8, 158.2, 156.6, 152.6, 150.0, 147.2, 146.5, 142.1, 138.6, 138.4, 134.3, 133.1, 130.4, 129.8, 128.7, 127.8, 127.6, 127.4, 127.0, 126.5, 123.7, 121.0, 114.9, 113.8, 109.4, 68.2, 68.1, 67.3, 56.2, 47.7, 44.4, 40.7, 37.1, 34.7, 32.1, 30.6, 30.6, 29.7, 29.5, 29.3, 29.2, 28.7, 27.1, 26.4, 26.2, 26.0, 25.5, 25.4, 24.0, 22.9, 14.3.

IR (ATR): 2925, 2851 cm^{-1}.

HRMS (MALDI-TOF) calcd. for $C_{193}H_{221}N_2O_6$ ($[M+H]^+$): 2662.7044, found 2662.7027.

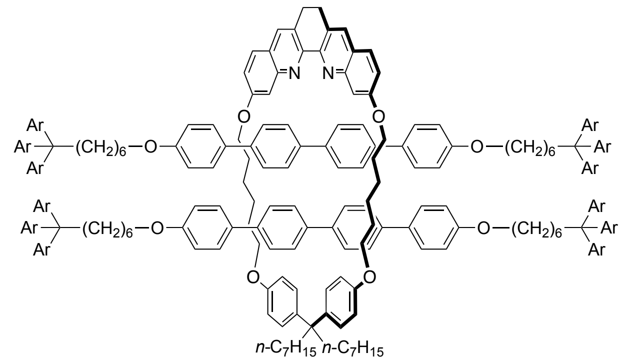

3Bb

3Bb

2b (65.8 mg, 60 μmol) was taken, and the mixture was stirred for 14 h to give a pale-yellow solid. Yield: 25.9 mg (5.4 μmol, 54%).

mp: 132.1-134.2 °C

^{1}H NMR (500 MHz, $CDCl_3$): δ 7.74 (s, 2H), 7.54 (d, J = 9.2 Hz, 2H), 7.50-7.37 (m, 74H), 7.25-7.23 (m, 24H), 7.20 (d, J = 8.0 Hz, 24H), 7.08-7.10 (2H), 6.98 (d, J = 9.2 Hz, 4H), 6.82 (d, J = 8.6 Hz, 8H), 6.65 (d, J = 9.2 Hz, 4H), 3.81-3.75 (m, 8H), 3.71 (t, J = 6.3 Hz, 8H), 2.97 (s, 4H), 2.49-2.43 (m, 20H), 1.94-1.81 (m, 56H), 1.74-1.68 (m, 18H), 1.48-1.36 (m, 62H), 1.27-1.07 (m, 52H), 0.89-0.79 (m, 10H).

^{13}C NMR (125 MHz, $CDCl_3$): δ 160.0, 158.9, 156.8, 153.1, 150.2, 147.2, 146.5, 141.9, 139.6, 138.7, 138.5, 138.4, 133.9, 132.6, 130.6, 129.8, 128.6, 128.0, 127.9, 127.4, 127.2, 127.0, 126.4, 123.7, 120.4, 115.1, 113.9, 109.4, 68.2, 67.3, 56.1, 47.5, 44.4, 40.6, 36.9, 34.7, 32.1, 31.8, 31.2, 30.6, 30.5, 29.7, 29.5, 28.8, 27.1, 26.4, 26.03, 25.97, 25.6, 24.0, 22.9, 14.3 (three signals are missing).

IR (ATR): 2924, 2850 cm^{-1}.

HRMS (MALDI-TOF) calcd. for $C_{351}H_{383}N_2O_8$ ($[M+H]^+$): 4753.9619, found 4753.9568.

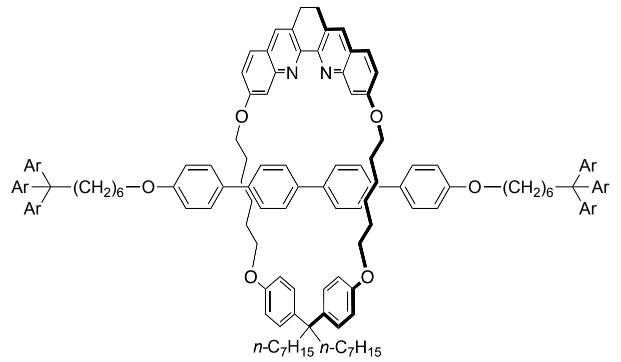

4Bb

4Bb

Pale-yellow solid. Yield: 4.9 mg (1.7 μmol, 17%).

mp: 114.3-114.8 °C

^{1}H NMR (500 MHz, $CDCl_3$): δ 7.87 (s, 2H), 7.60-7.46 (m, 40H), 7.36 (d, *J* = 8.6 Hz, 12H), 7.25-7.24 (m, 12H), 7.12 (dd, *J* = 9.2, 2.3 Hz, 2H), 7.04 (d, *J* = 8.6 Hz, 4H), 6.86 (d, *J* = 8.6 Hz, 4H), 6.66 (d, *J* = 8.6 Hz, 4H), 3.86-3.80 (m, 8H), 3.74 (t, *J* = 6.6 Hz, 4H), 3.13 (s, 4H), 2.62-2.49 (m, 10H), 2.01-1.64 (m, 52H), 1.48-1.17 (m, 60H), 0.95 (brs, 4H), 0.83 (t, *J* = 6.9 Hz, 6H).

^{13}C NMR (125 MHz, $CDCl_3$): δ 159.8, 158.9, 156.6, 152.7, 150.0, 147.2, 146.5, 142.2, 139.6, 138.8, 138.6, 138.4, 134.2, 132.9, 130.4, 129.8, 128.8, 128.1, 127.8, 127.4, 127.3, 127.0, 126.5, 123.7, 121.0, 115.0, 113.9, 109.3, 68.13, 68.05, 67.3, 56.2, 47.7, 44.4, 40.7, 37.1, 34.7, 32.1, 30.6, 30.5, 29.6, 29.4, 29.2, 29.1, 28.7, 27.1, 26.4, 26.2, 26.0, 25.5, 25.4, 24.0, 22.9, 14.3 (one signal is missing).

IR (ATR): 2926, 2852 cm^{-1}.

HRMS (MALDI-TOF) calcd. for $C_{205}H_{229}N_2O_6$ ($[M+H]^+$): 2814.7670, found 2814.7691.

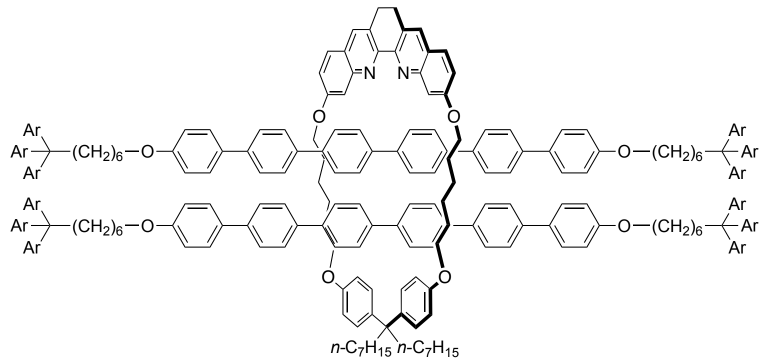

3Bc

3Bc

2c (65.6 mg, 60 μmol) was taken, and the mixture was stirred for 16 h to give a pale-yellow solid. Yield: 24.8 mg (4.9 μmol, 49%).

mp: 170.0-171.9 °C

^{1}H NMR (500 MHz, $CDCl_3$): δ 7.79 (s, 2H), 7.56-7.51 (m, 26H), 7.47-7.42 (m, 58H), 7.38 (d, *J* = 8.6 Hz, 8H), 7.27-7.25 (m, 24H), 7.21 (d, *J* = 8.0 Hz, 24H), 7.09 (d, *J* = 8.6 Hz, 2H), 7.05 (d, *J* = 8.0 Hz, 4H), 6.80 (d, *J* = 8.6 Hz, 8H), 6.68 (d, *J* = 8.6 Hz, 4H), 3.78-3.69 (m, 16H), 3.03 (s, 4H), 2.51-2.47 (m, 20H), 2.02-1.96 (m, 4H), 1.89-1.63 (m, 70H), 1.54-1.50 (m, 4H), 1.45-1.06 (m, 110H), 0.92 (brs, 4H), 0.82 (t, *J* = 7.2 Hz, 6H).

^{13}C NMR (125 MHz, $CDCl_3$): δ 159.9, 158.9, 156.8, 153.2, 150.2, 147.2, 146.5, 141.9, 139.7, 139.5, 139.2, 138.6, 138.44, 138.37, 134.0, 132.5, 130.6, 129.8, 128.6, 128.0, 127.9, 127.4, 127.3, 127.0, 126.4, 123.7, 120.3, 115.0, 114.0, 109.5, 68.1, 67.4, 56.1, 47.5, 44.4, 40.6, 34.7, 32.1, 30.6, 30.5, 29.7, 29.6, 29.5, 28.8, 27.1, 26.4, 26.1, 26.0, 25.71, 25.68, 24.0, 22.9, 14.3 (six signals are missing).

IR (ATR): 2925, 2851 cm^{-1}.

HRMS (MALDI-TOF) calcd. for $C_{375}H_{399}N_2O_8$ ($[M+H]^+$): 5058.0871, found 5058.0816.

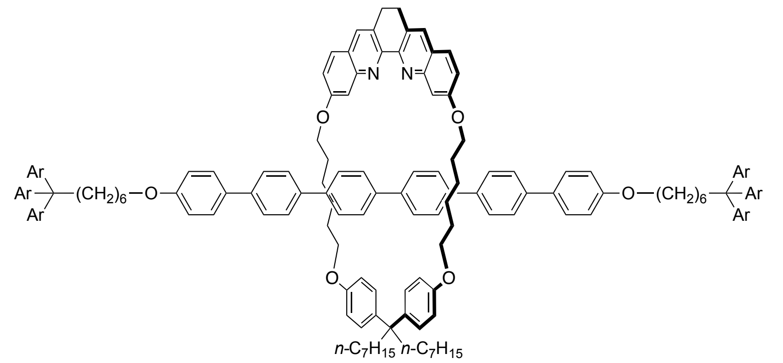

4Bc

4Bc

Pale-yellow solid. Yield: 6.1 mg (2.1 μmol, 21%).

mp: 146.1-147.5°C

^{1}H NMR (500 MHz, $CDCl_3$): δ 7.89 (s, 2H), 7.64-7.50 (m, 48H), 7.37 (d, *J* = 8.6 Hz, 12H), 7.26-7.24 (m, 12H), 7.12 (dd, *J* = 8.9, 2.0 Hz, 2H), 7.08 (d, *J* = 8.9 Hz, 4H), 6.89 (d, *J* = 8.6 Hz, 4H), 6.68 (d, *J* = 8.6 Hz, 4H), 3.88-3.81 (m, 8H), 3.73 (t, *J* = 6.6 Hz, 4H), 3.15 (s, 4H), 2.64-2.50 (m, 10H), 2.04-1.60 (m, 50H), 1.49-1.19 (m, 62H), 0.97 (brs, 4H), 0.83 (t, *J* = 7.2 Hz, 6H).

^{13}C NMR (125 MHz, $CDCl_3$): δ 159.8, 158.9, 156.7, 152.6, 150.0, 147.2, 146.5, 142.3, 139.8, 139.6, 139.4, 138.7, 138.6, 138.4, 134.2, 132.9, 130.4, 129.8, 128.8, 128.1, 127.8, 127.4, 127.1, 127.0, 126.5, 123.7, 121.0, 115.0, 113.9, 109.3, 68.1, 68.0, 67.4, 56.2, 47.8, 44.4, 40.7, 37.1, 34.7, 32.1, 30.6, 30.4, 29.5, 29.4, 29.14, 29.11, 28.7, 27.1, 26.4, 26.2, 25.9, 25.5, 25.4, 24.0, 22.9, 14.3 (three signals are missing).

IR (ATR): 2926, 2851 cm^{-1}.

HRMS (MALDI-TOF) calcd. for $C_{217}H_{237}N_2O_6$ ($[M+H]^+$): 2966.8296, found 2966.8337.

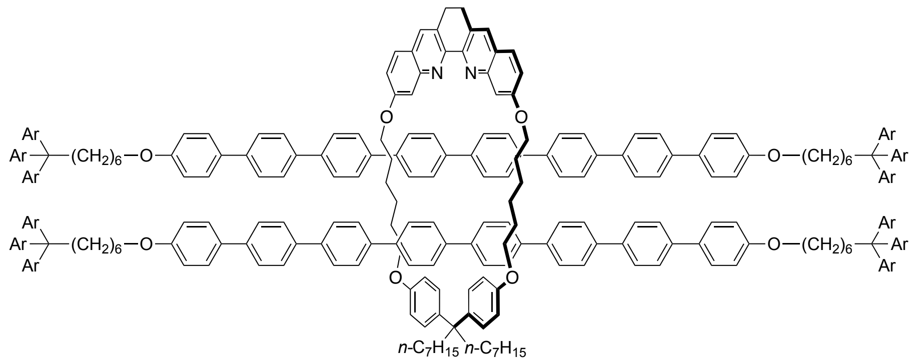

3Bd

3Bd

2d (75.0 mg, 60 μmol) was taken, and the mixture was stirred for 11 h to give a pale-yellow solid. Yield: 21.8 mg (4.1 μmol, 41%).

mp: 133.4-136.1 °C

^{1}H NMR (500 MHz, $CDCl_3$): δ 7.81 (s, 2H), 7.57-7.42 (m, 100H), 7.37 (d, *J* = 6.9 Hz, 8H), 7.27 (d, *J* = 8.6 Hz, 24H), 7.20 (d, *J* = 8.0 Hz, 24H), 7.10-7.06 (m, 6H), 6.77 (d, *J* = 8.6 Hz, 8H), 6.69 (d, *J* = 8.6 Hz, 4H), 3.76-3.68 (m, 16H), 3.06 (s, 4H), 2.49-2.46 (m, 20H), 2.03-2.00 (m, 4H), 1.88-1.80 (m, 52H), 1.74-1.71 (m, 14H), 1.63-1.57 (m, 4H), 1.45-1.06 (m, 114H), 0.95-0.93 (m, 4H), 0.81 (t, *J* = 7.2 Hz, 6H).

^{13}C NMR (125 MHz, $CDCl_3$): δ 160.0, 159.0, 156.8, 153.3, 150.2, 147.2, 147.1, 146.5, 141.9, 139.3, 138.9, 138.6, 138.5, 138.4, 138.3, 137.7, 134.0, 132.0, 131.5, 130.6, 129.7, 128.6, 128.0, 127.84, 127.76, 127.3, 127.1, 127.0, 126.8, 126.4, 123.7, 120.3, 115.2, 115.1, 114.0, 109.4, 68.0, 67.3, 56.1, 47.5, 44.4, 40.5, 36.9, 34.7, 32.1, 30.7, 30.4, 29.6, 29.5, 28.7, 27.1, 26.4, 26.1, 25.8, 25.7, 25.6, 24.0, 22.9, 14.3 (four signals are missing).

IR (ATR): 2925, 2851 cm^{-1}.

HRMS (MALDI-TOF) calcd. for $C_{399}H_{415}N_2O_8$ ($[M+H]^+$): 5362.2123, found 5362.2040.

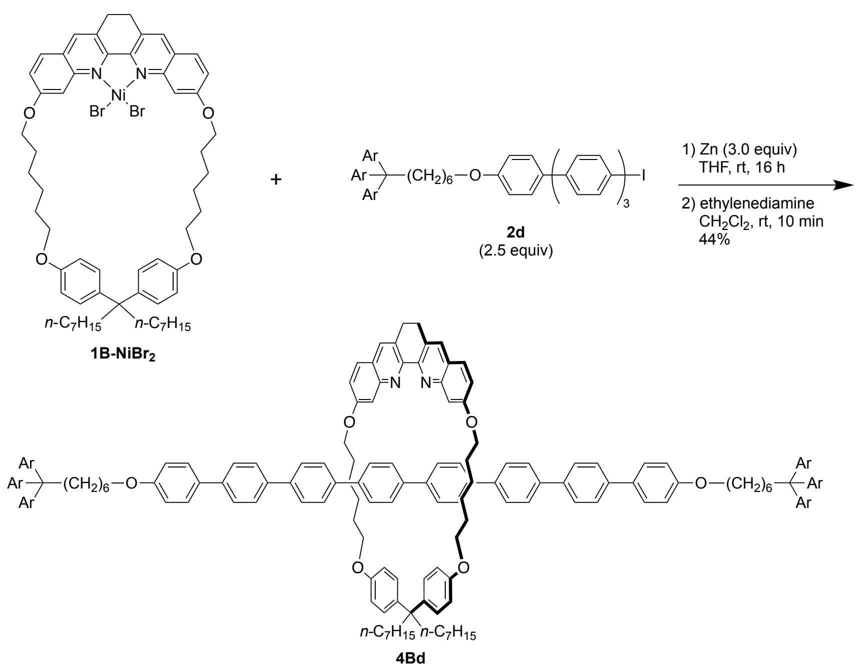

4Bd

To a suspension of **1B-NiBr₂** (22 mg, 20 μmol) in dry THF (1.0 mL) was added Zn powder (3.9 mg, 60 μmol) at rt under Ar, and the mixture was sonicated for 3 min. To the dark green mixture was added **2d** (63 mg, 50 μmol) and the resulting mixture was stirred at rt for 16 h. To the mixture CH_2Cl_2 (2.0 mL) and ethylenediamine (2.0 mL) were added and the mixture was stirred at rt for 10 min. The resulting mixture was extracted with CH_2Cl_2. The organic layer was washed with brine, dried over anhydrous Na_2SO_4 and concentrated *in vacuo*. The crude mixture was purified by flash column chromatography over silica gel (hexane : CH_2Cl_2 = 3 : 1, then CH_2Cl_2 : CH_3OH = 20 : 1) and gel permeation chromatography to give a pale-yellow solid. Yield: 27.3 mg (8.8 μmol, 44%).

mp: 160.7-161.8 °C

^{1}H NMR (500 MHz, $CDCl_3$): δ 7.90 (s, 2H), 7.68-7.50 (m, 56H), 7.37 (d, *J* = 8.6 Hz, 12H), 7.26-7.24 (m, 12H), 7.13-7.09 (m, 6H), 6.91 (d, *J* = 8.6 Hz, 4H), 6.69 (d, *J* = 8.6 Hz, 4H), 3.90-3.72 (m, 12H), 3.16 (s, 4H), 2.65-2.50 (m, 10H), 2.05-1.62 (m, 50H), 1.49-1.20 (m, 62H), 0.98 (m, 4H), 0.83 (t, *J* = 7.2 Hz, 6H).

^{13}C NMR (125 MHz, $CDCl_3$): δ 159.8, 158.9, 156.7, 152.7, 150.0, 147.3, 146.5, 142.3, 139.9, 139.7, 139.62, 139.55, 139.4, 138.8, 138.6, 138.4, 134.2, 133.0, 130.4, 129.8, 128.8, 128.1, 127.8, 127.5, 127.4, 127.2, 127.0, 126.5, 123.7, 121.0, 115.1, 113.9, 109.3, 68.2, 68.0, 67.4, 56.2, 47.8, 44.4, 40.7, 37.2, 34.7, 32.1, 30.6, 30.4, 29.5, 29.4, 29.1, 28.7, 27.1, 26.4, 26.2, 25.9, 25.5, 25.4, 24.1, 22.9, 14.3 (five signals are missing).

IR (ATR): 2926, 2851 cm^{-1}.

HRMS (MALDI-TOF) calcd. for $C_{229}H_{245}N_2O_6$ ($[M+H]^+$): 3118.8922, found 3118.8798.

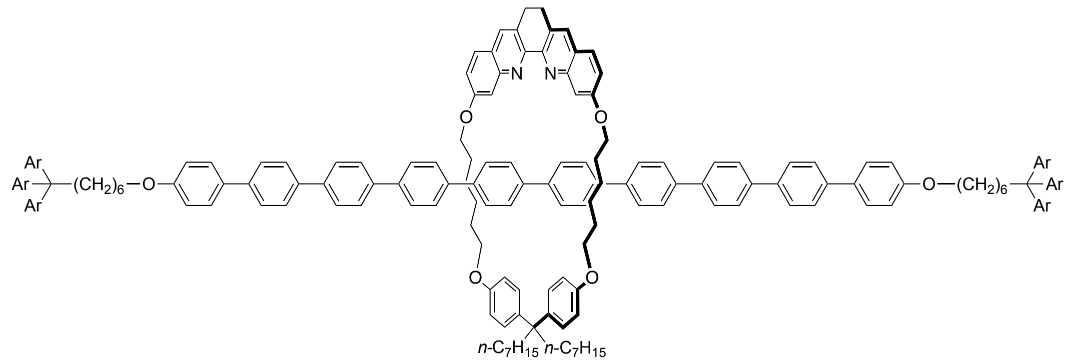

4Be

4Be

2e (79.5 mg, 60 μmol) was taken, and the mixture was stirred for 15 h to give a pale-yellow solid. Yield: 20.3 mg (6.2 μmol, 62%).

mp: 180.2-181.2 °C

^{1}H NMR (500 MHz, $CDCl_3$): δ 7.90 (s, 2H), 7.68-7.55 (m, 40H), 7.51-7.48 (m, 24H), 7.35 (d, J = 8.6 Hz, 12H), 7.24-7.23 (m, 12H), 7.11-7.07 (m, 6H), 6.90 (d, J = 8.6 Hz, 4H), 6.67 (d, J = 8.6 Hz, 4H), 3.89 (t, J = 6.3 Hz, 4H), 3.77 (t, J = 7.2 Hz, 4H), 3.70 (t, J = 6.6 Hz, 4H), 3.17 (s, 4H), 2.64-2.49 (m, 10H), 2.02-1.58 (m, 50H), 1.47-1.17 (m, 62H), 0.96 (brs, 4H), 0.81 (t, J = 7.2 Hz, 6H).

^{13}C NMR (125 MHz, $CDCl_3$): δ 159.8, 159.0, 156.7, 152.7, 150.0, 147.3, 146.5, 142.3, 140.0, 139.8, 139.6, 139.5, 138.8, 138.6, 138.4, 134.2, 133.0, 130.5, 129.8, 128.9, 128.1, 127.8, 127.6, 127.45, 127.39, 127.2, 127.0, 126.5, 123.8, 121.0, 115.1, 113.9, 109.2, 68.2, 67.9, 67.4, 56.2, 47.8, 44.4, 40.7, 37.2, 34.7, 32.1, 30.6, 30.4, 29.5, 29.4, 29.1, 28.7, 27.1, 26.4, 26.1, 25.9, 25.5, 25.4, 24.1, 22.9, 14.3 (nine signals are missing).

IR (ATR): 2925, 2851 cm^{-1}.

HRMS (MALDI-TOF) calcd. for $C_{241}H_{253}N_2O_6$ ($[M+H]^+$): 3270.9548, found 3270.9594.

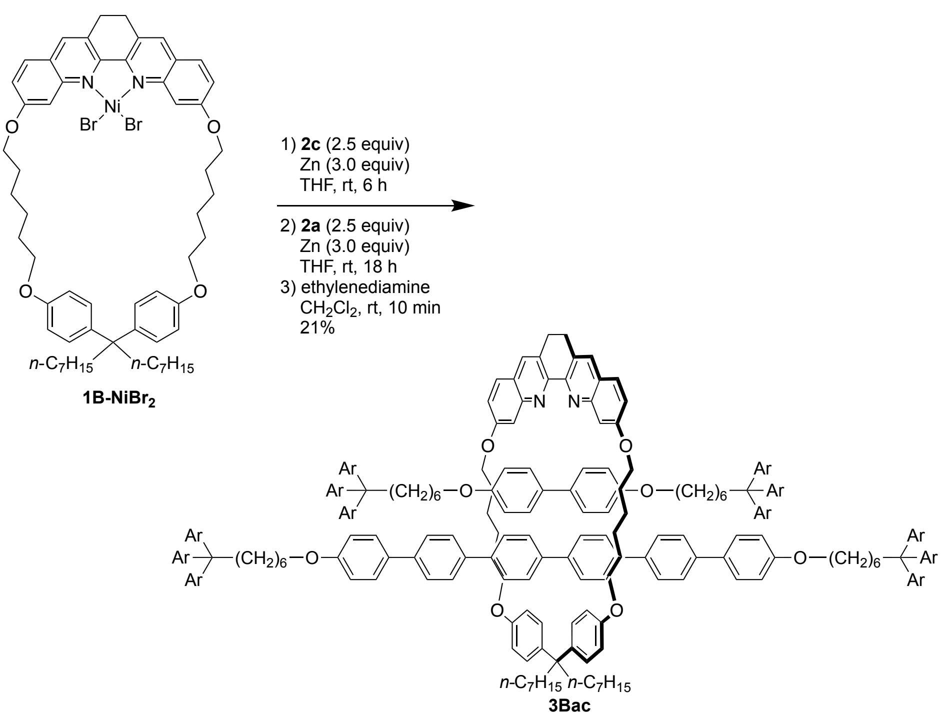

3Bac

To a suspension of **1B-NiBr$_2$** (11 mg, 10 μmol) in dry THF (0.5 mL) was added Zn powder (2.0 mg, 30 μmol) at rt under Ar, and the mixture was sonicated for 3 min. To the dark green mixture was added **2c** (29 mg, 25 μmol) and the resulting mixture was stirred at rt for 6 h. To the mixture **2a** (26 mg, 25 μmol) and Zn powder (2.0 mg, 30 μmol) were added, and the resulting mixture was stirred at rt for 18 h. To the mixture were added CH_2Cl_2 (2.0 mL) and ethylenediamine (2.0 mL), and the mixture was stirred at rt for 10 min. The resulting mixture was extracted with CH_2Cl_2. The organic layer was washed with brine, dried over anhydrous Na_2SO_4 and concentrated *in vacuo*. The crude mixture was purified by flash column chromatography over silica gel (hexane : CH_2Cl_2 = 4 : 1, then hexane : CH_2Cl_2 = 2 : 1, then CH_2Cl_2 : EtOAc = 2 : 1) and gel permeation chromatography to give a pale-yellow solid. Yield: 10.1 mg (2.1 μmol, 21%).

mp: 157.5-158.9 °C

^{1}H NMR (500 MHz, $CDCl_3$): δ 7.74 (s, 2H), 7.63-7.42 (m, 64H), 7.38 (d, *J* = 8.6 Hz, 12H), 7.30-7.26 (m, 12H), 7.22-7.14 (m, 36H), 7.08 (dd, *J* = 9.2, 2.3 Hz, 2H), 6.97 (d, *J* = 9.2 Hz, 4H), 6.87 (d, *J* = 8.6 Hz, 4H), 6.65-6.63 (m, 8H), 3.79-3.75 (m, 12H), 3.53 (t, *J* = 6.9 Hz, 4H), 2.97 (s, 4H), 2.49-2.35 (m, 20H), 1.87-1.64 (m, 74H), 1.45-0.86 (m, 118H), 0.80 (t, *J* = 6.9 Hz, 6H).

^{13}C NMR (125 MHz, $CDCl_3$): δ 159.9, 159.0, 158.1, 156.8, 153.1, 150.2, 147.22, 147.17, 146.5, 141.9, 139.8, 139.6, 139.3, 138.6, 138.5, 138.40, 138.37, 133.9, 132.8, 132.7, 130.6, 129.8, 128.6, 128.1, 127.9, 127.43, 127.37, 127.35, 127.1, 127.01, 126.98, 126.43, 126.37, 123.7, 120.4, 115.1, 114.8, 113.9, 109.4, 68.2, 68.1, 67.3, 56.2, 56.1, 47.5, 44.4, 40.6, 40.5, 36.9, 34.7, 32.1, 30.6, 30.5, 30.4, 29.74, 29.65, 29.6, 29.5, 28.8, 27.1, 26.4, 26.0, 25.9, 25.6, 24.0, 22.9, 14.3 (fifteen signals are missing).

IR (ATR): 2923, 2850 cm^{-1}.

HRMS (MALDI-TOF) calcd. for $C_{351}H_{383}N_2O_8$ ($[M+H]^+$): 4753.9619, found 4753.9683.

3. ^{1}H NMR and ^{13}C NMR spectra

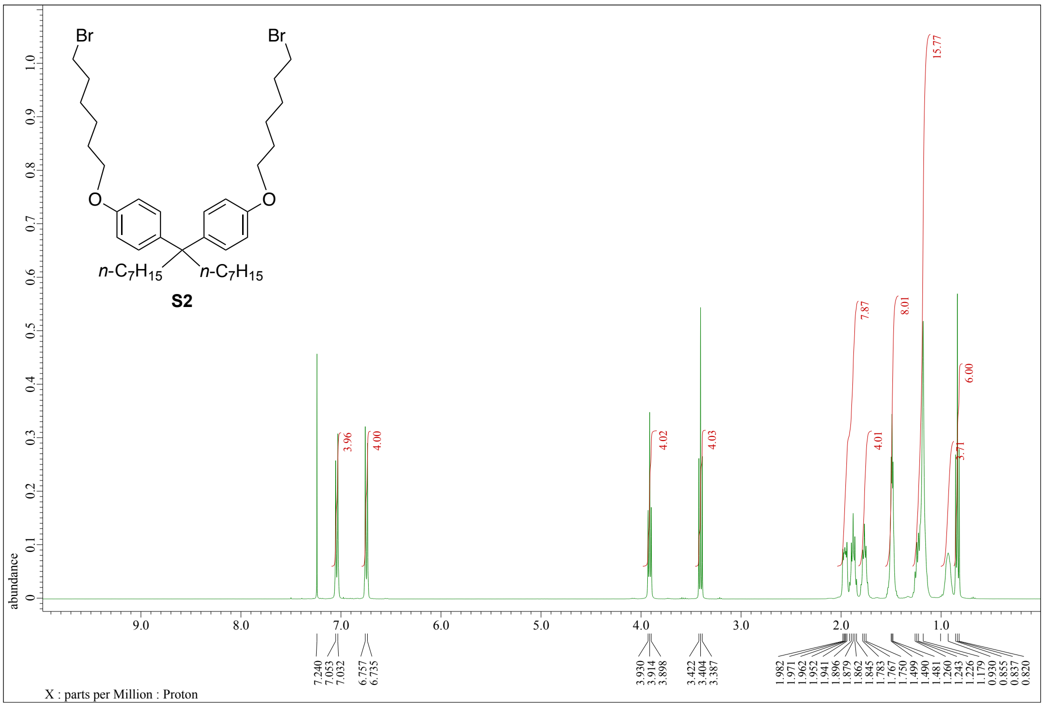

^{1}H NMR spectrum of **S2** (400 MHz, $CDCl_3$)

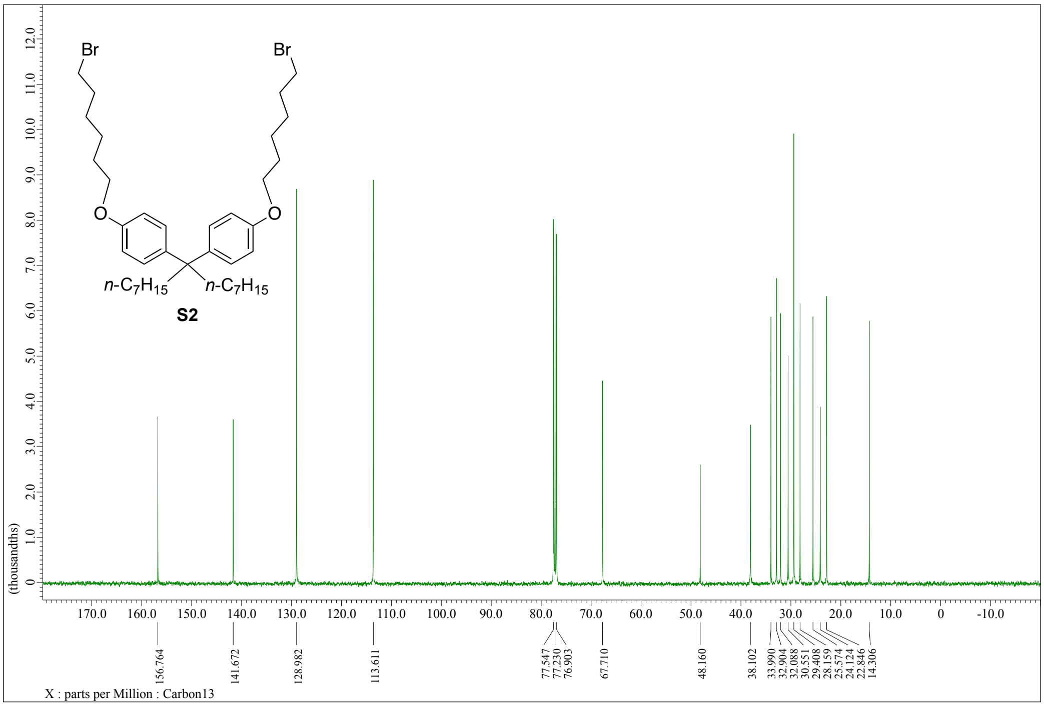

^{13}C NMR spectrum of **S2** (100 MHz, $CDCl_3$)

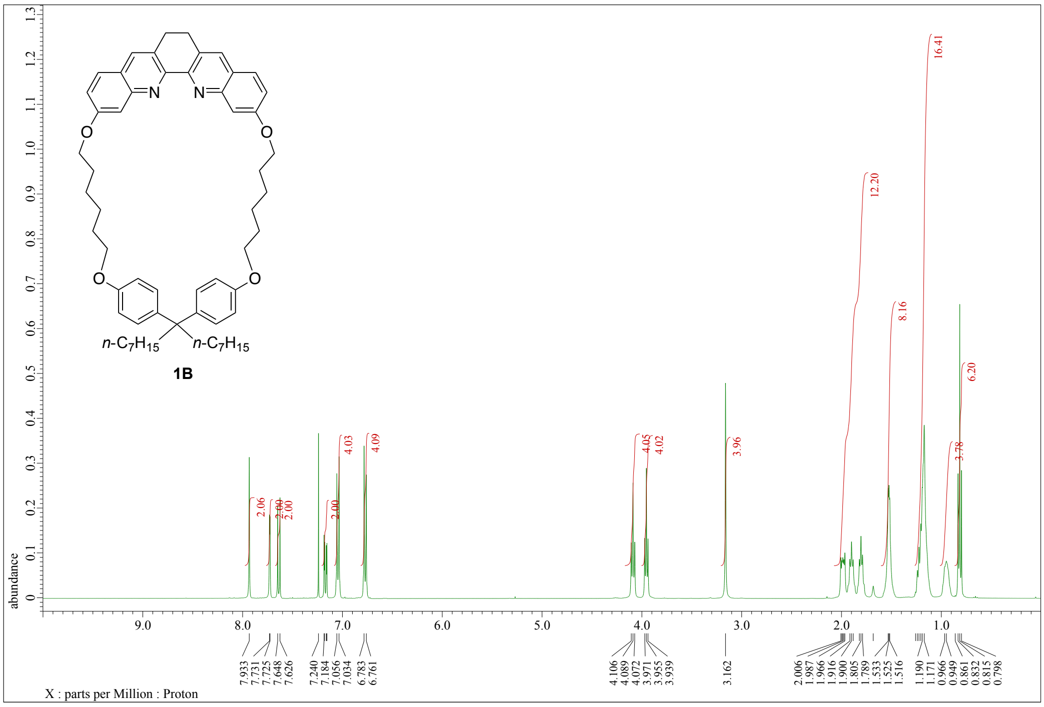

^{1}H NMR spectrum of **1B** (500 MHz, $CDCl_3$)

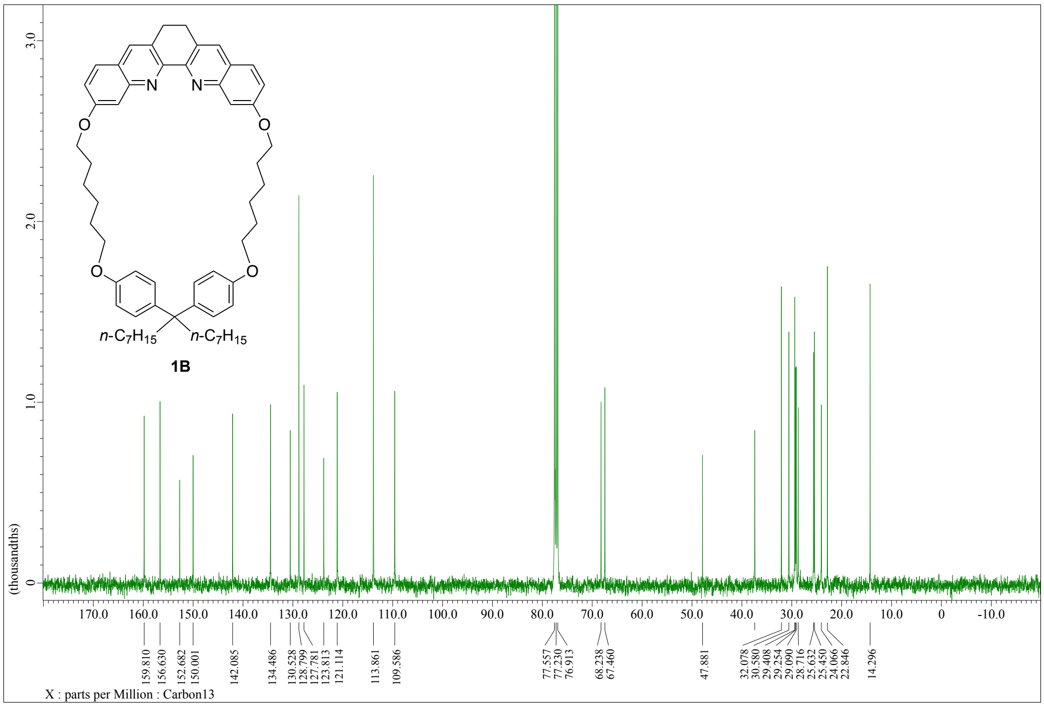

^{13}C NMR spectrum of **1B** (100 MHz, $CDCl_3$)

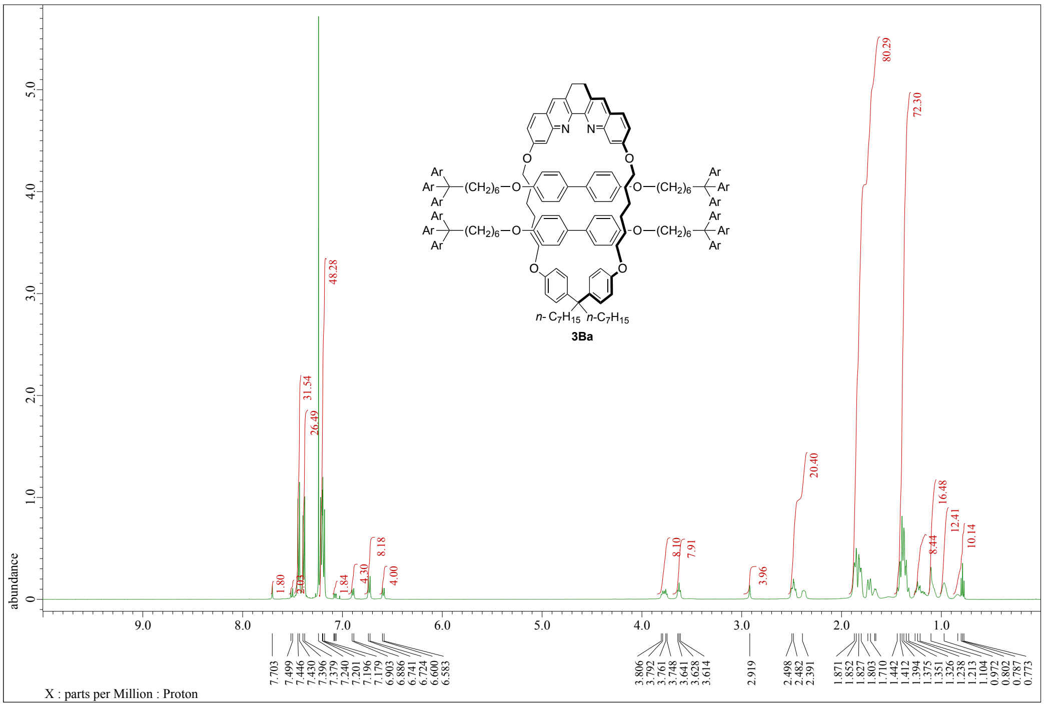

^{1}H NMR spectrum of **3Ba** (500 MHz, $CDCl_3$)

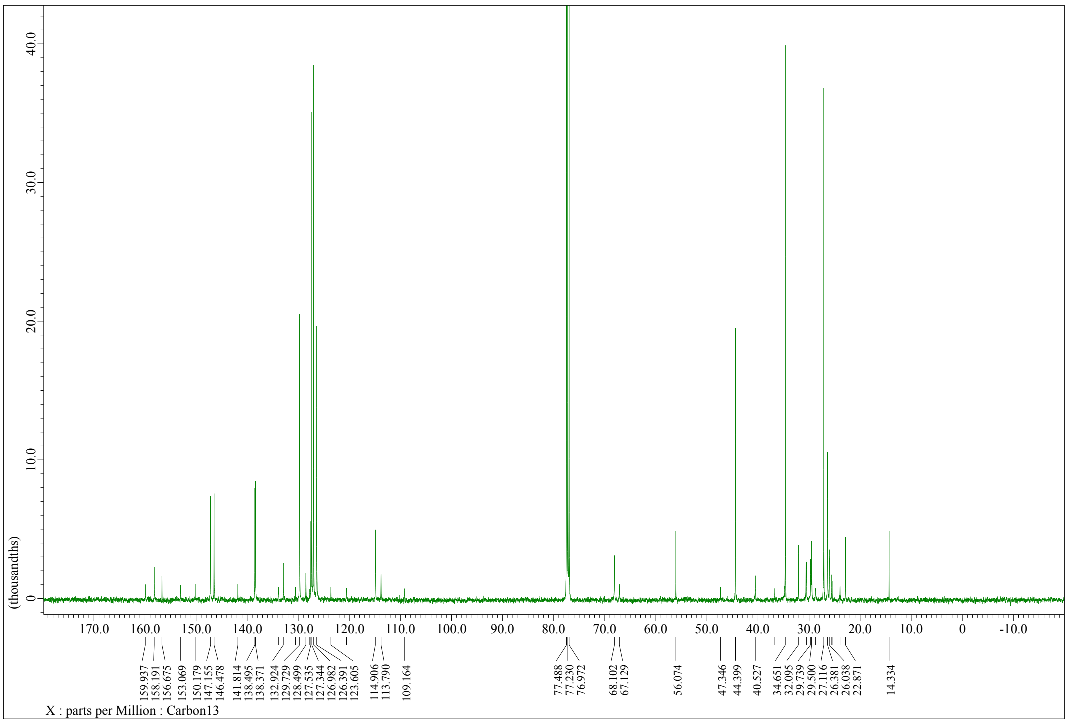

^{13}C NMR spectrum of **3Ba** (125 MHz, $CDCl_3$)

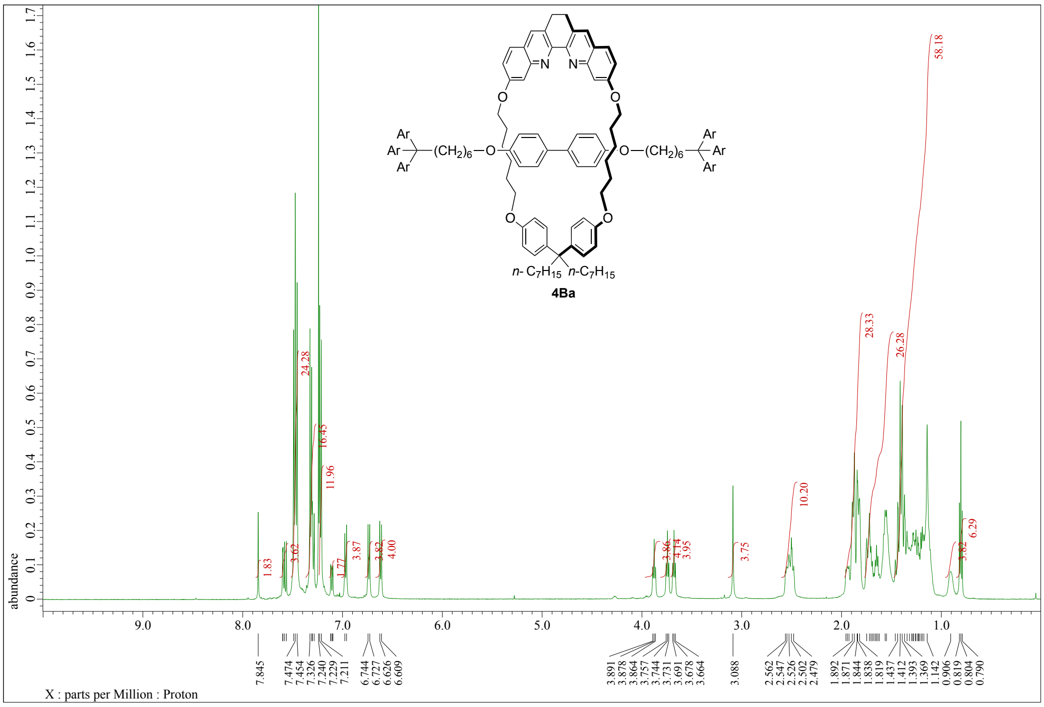

^{1}H NMR spectrum of **4Ba** (500 MHz, $CDCl_3$)

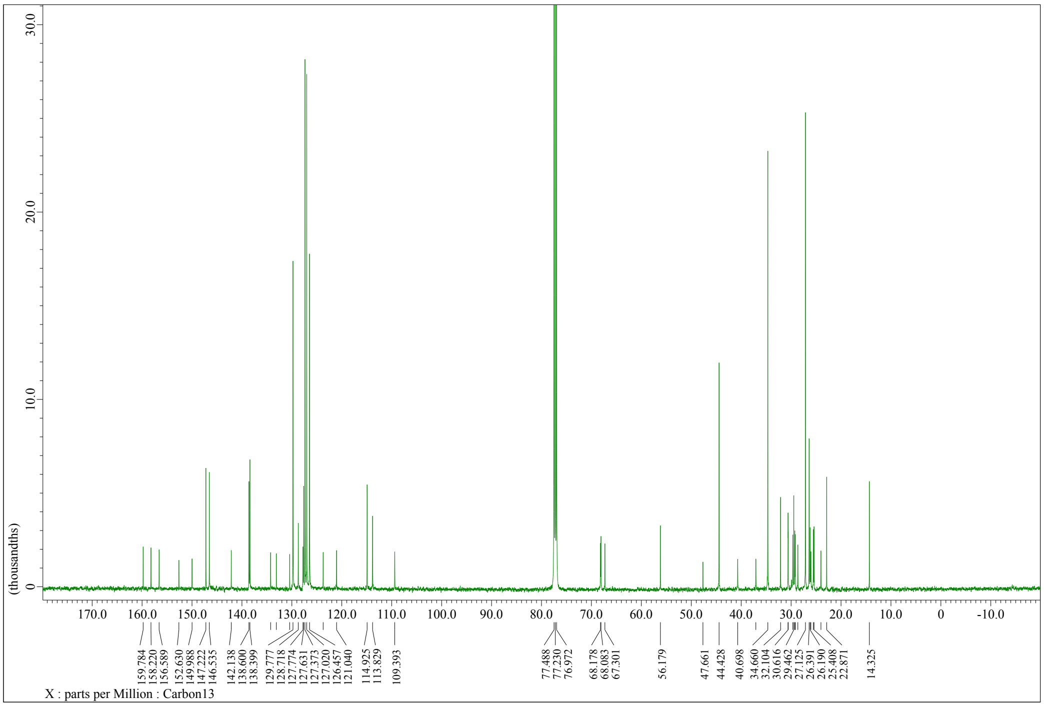

^{13}C NMR spectrum of **4Ba** (125 MHz, $CDCl_3$)

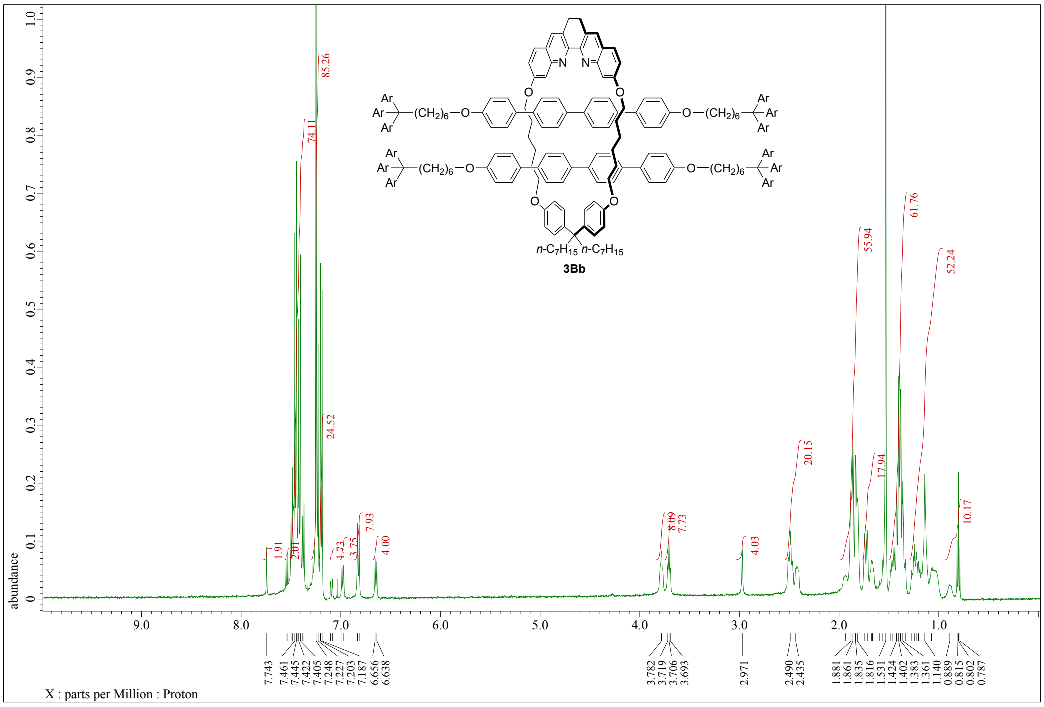

^{1}H NMR spectrum of **3Bb** (500 MHz, $CDCl_3$)

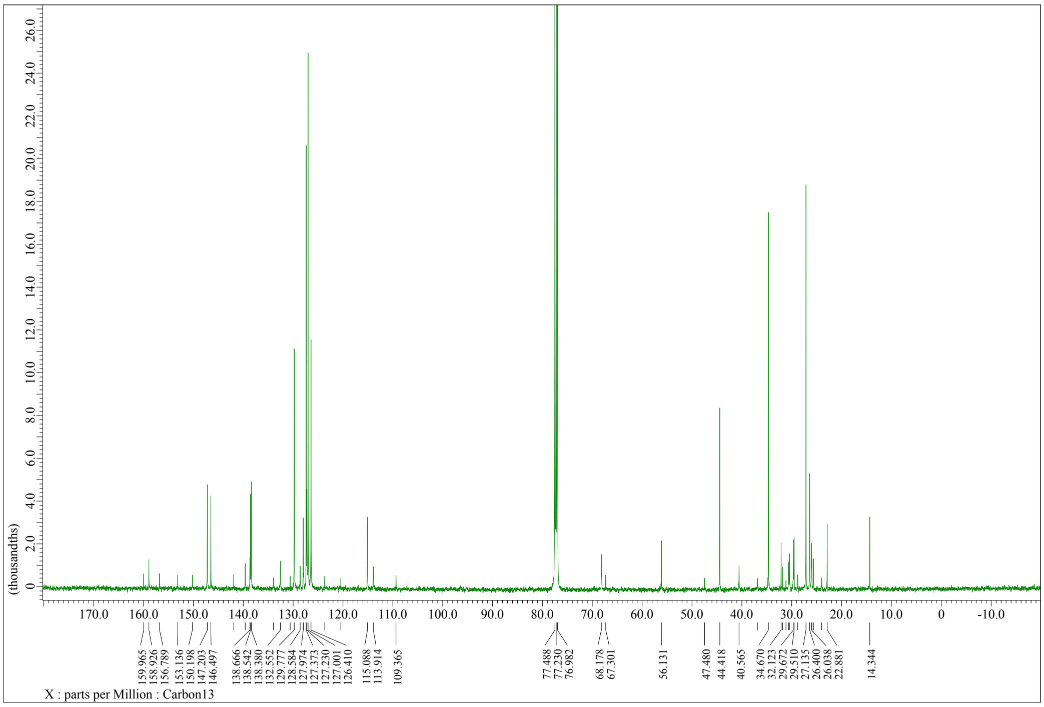

^{13}C NMR spectrum of **3Bb** (125 MHz, $CDCl_3$)

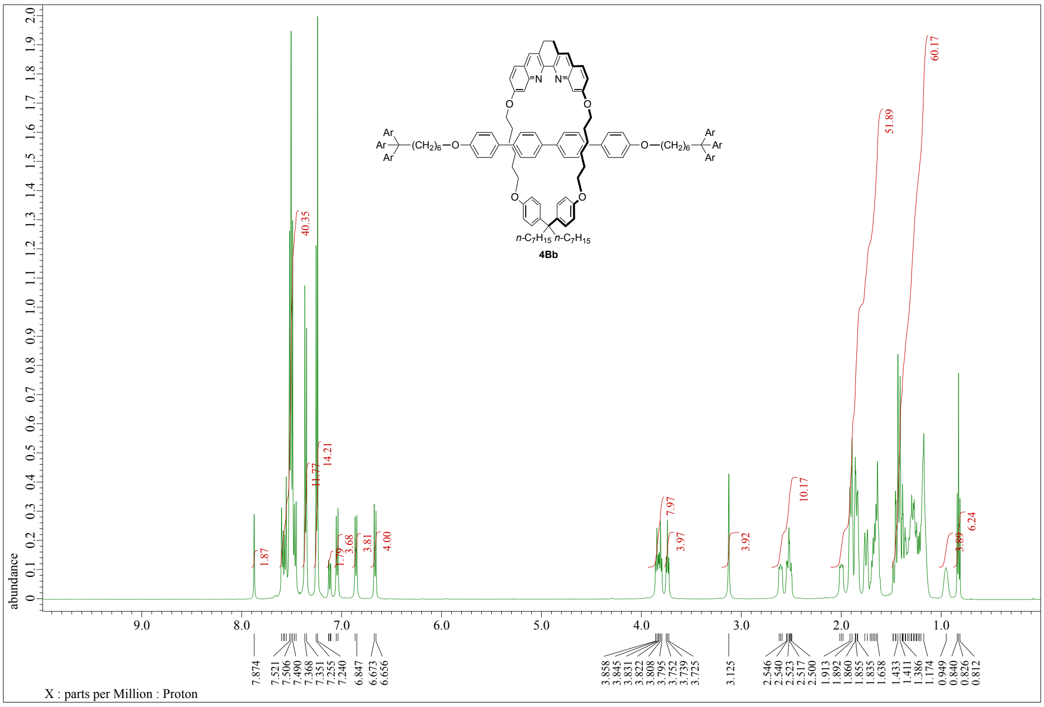

^{1}H NMR spectrum of **4Bb** (500 MHz, $CDCl_3$)

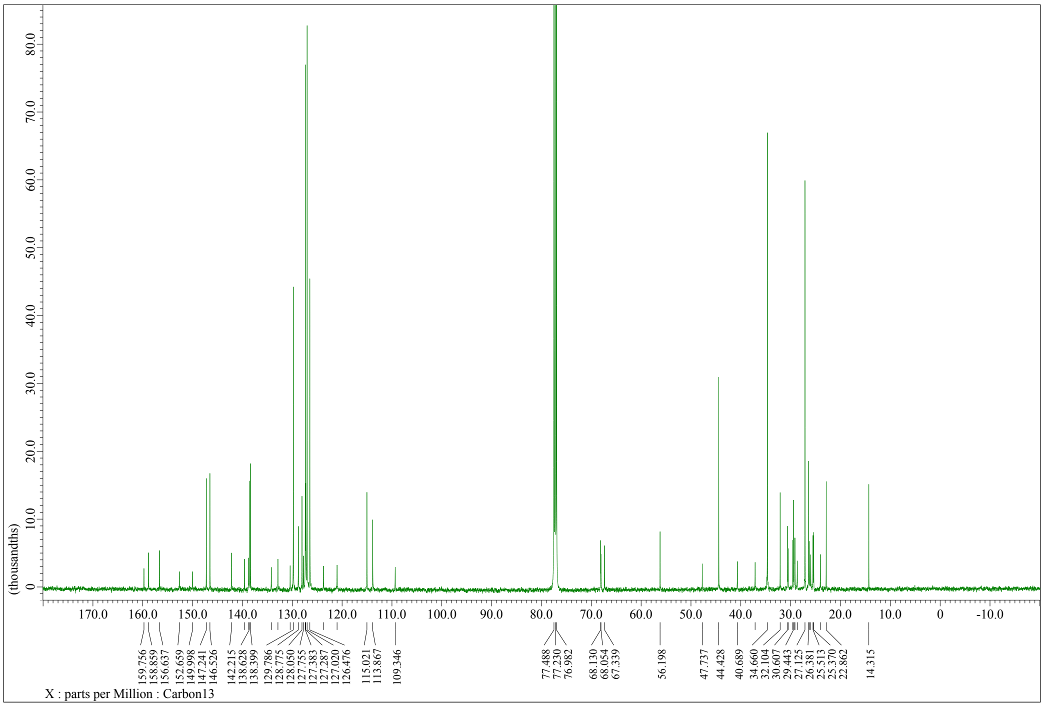

^{13}C NMR spectrum of **4Bb** (125 MHz, $CDCl_3$)

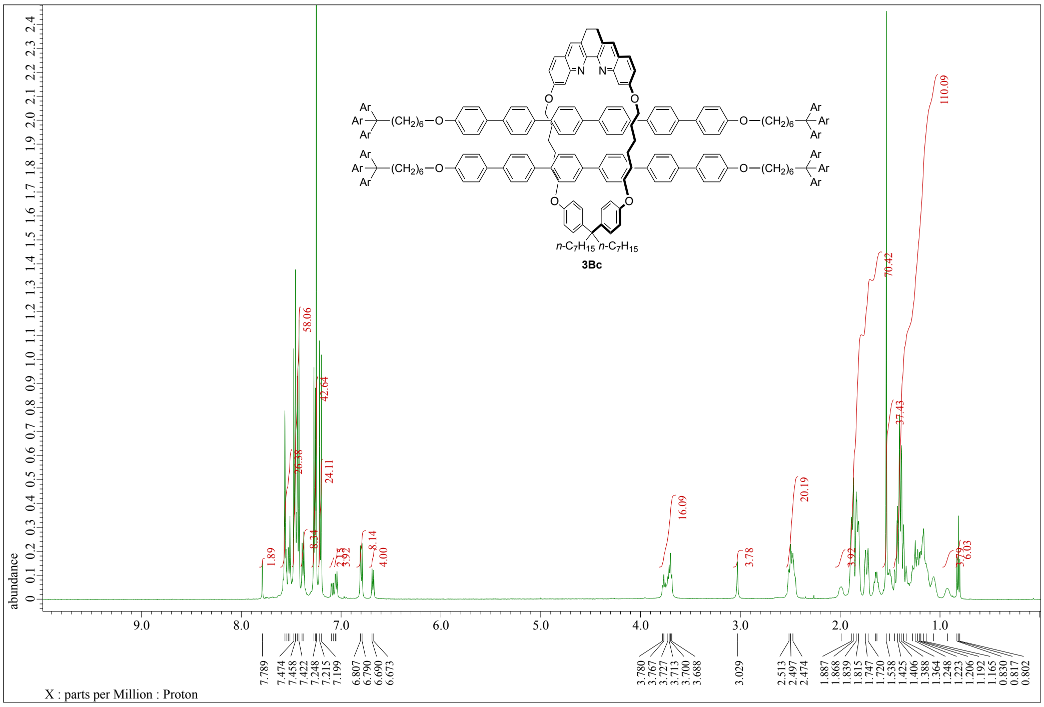

^{1}H NMR spectrum of **3Bc** (500 MHz, $CDCl_3$)

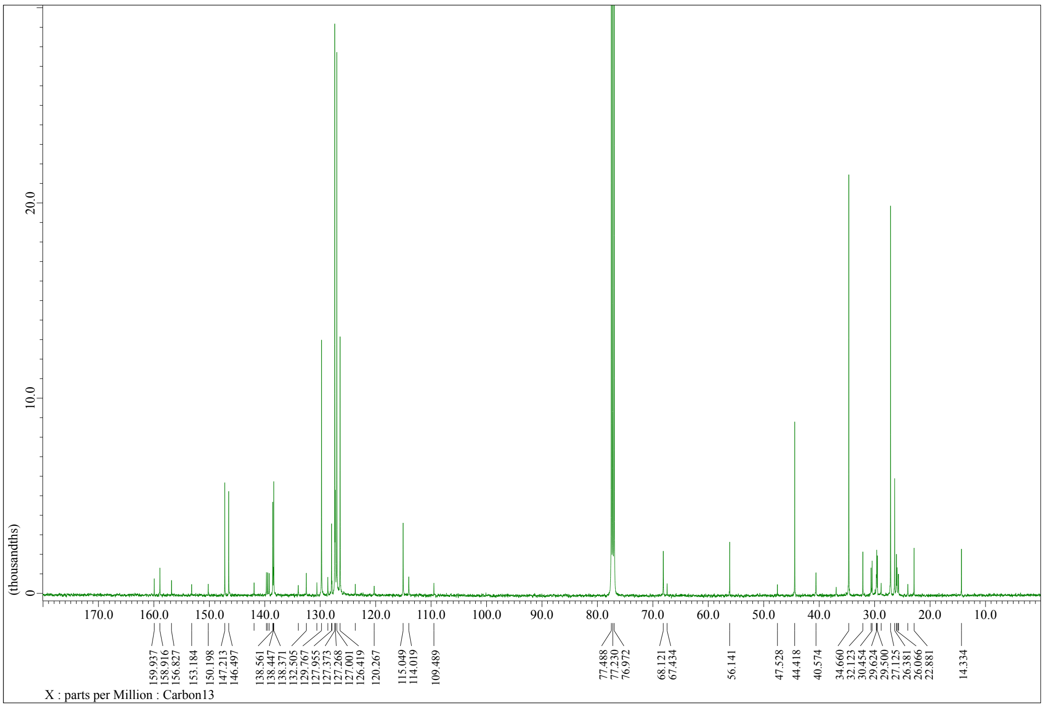

^{13}C NMR spectrum of **3Bc** (125 MHz, $CDCl_3$)

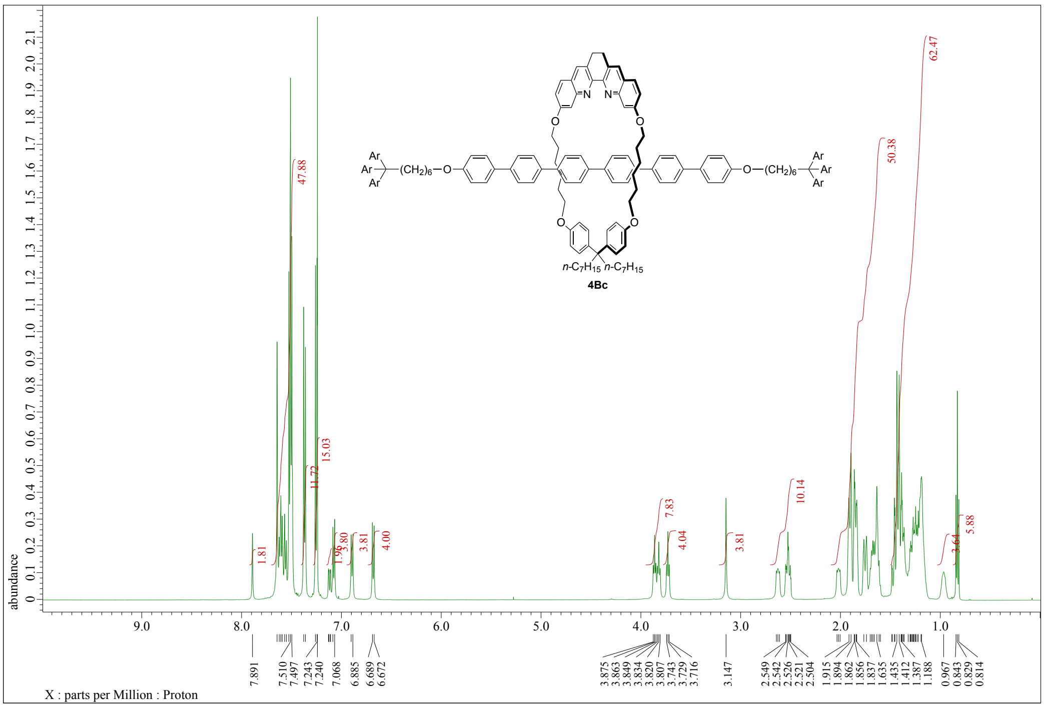

^{1}H NMR spectrum of **4Bc** (500 MHz, $CDCl_3$)

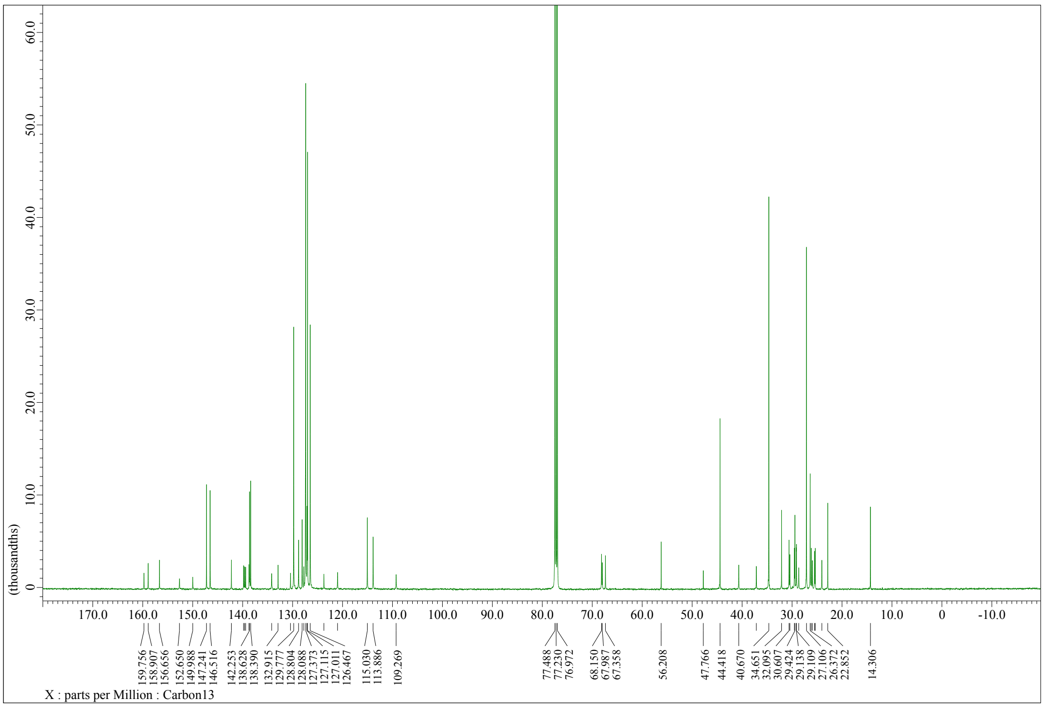

^{13}C NMR spectrum of **4Bc** (125 MHz, $CDCl_3$)

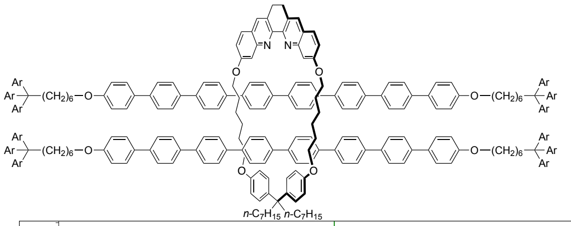

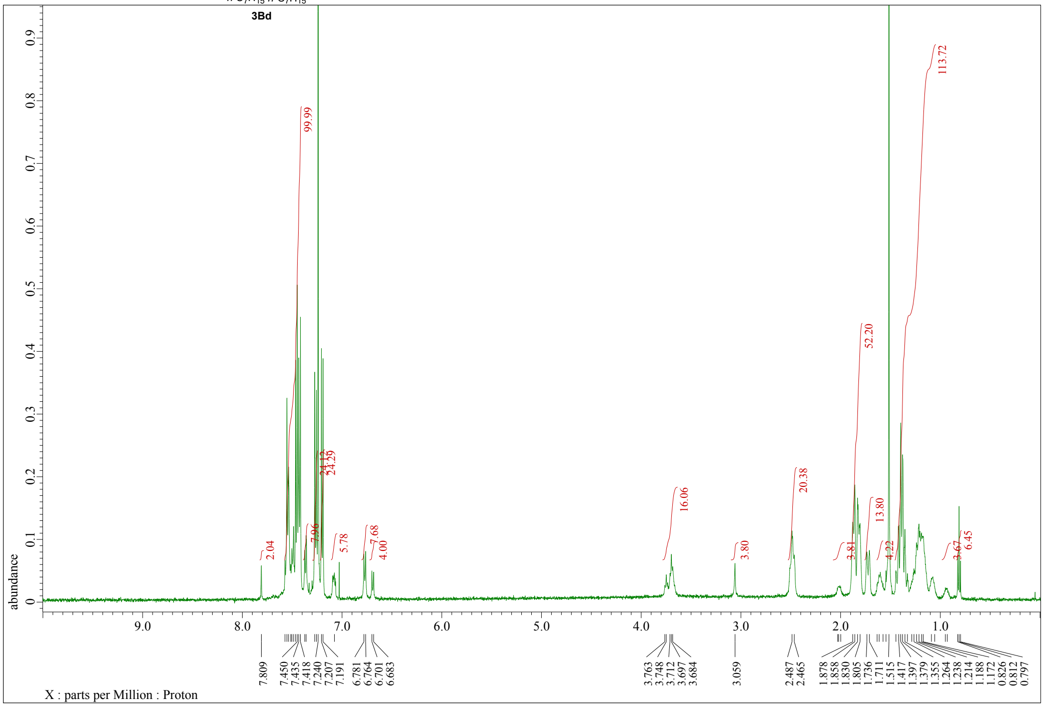

^{1}H NMR spectrum of **3Bd** (500 MHz, $CDCl_3$)

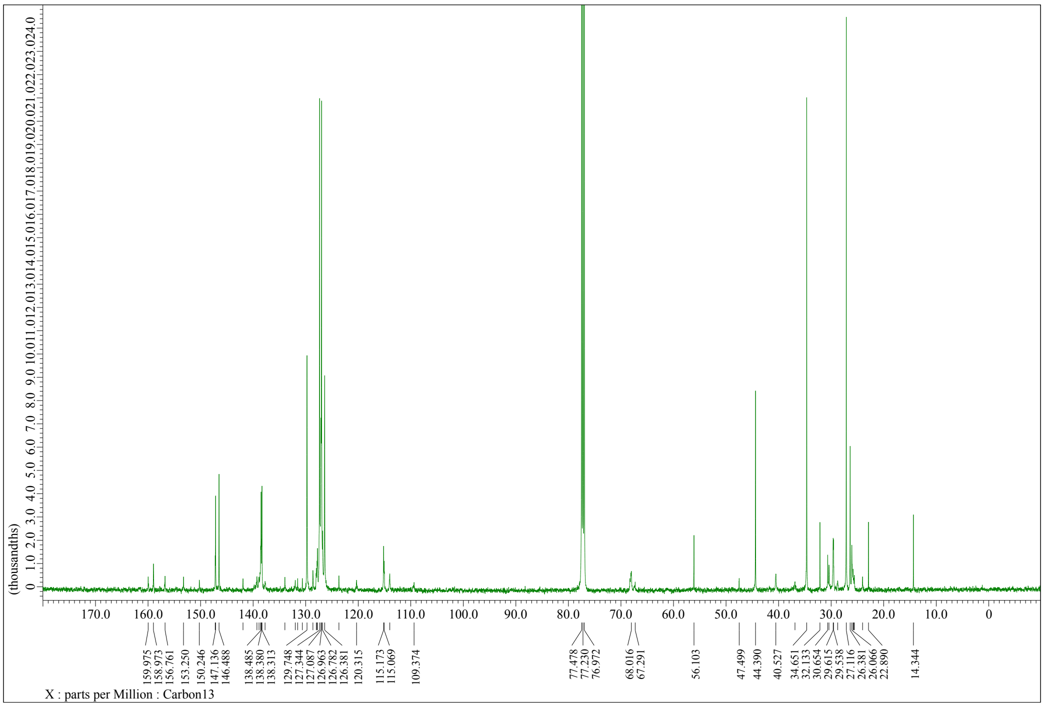

^{13}C NMR spectrum of **3Bd** (125 MHz, $CDCl_3$)

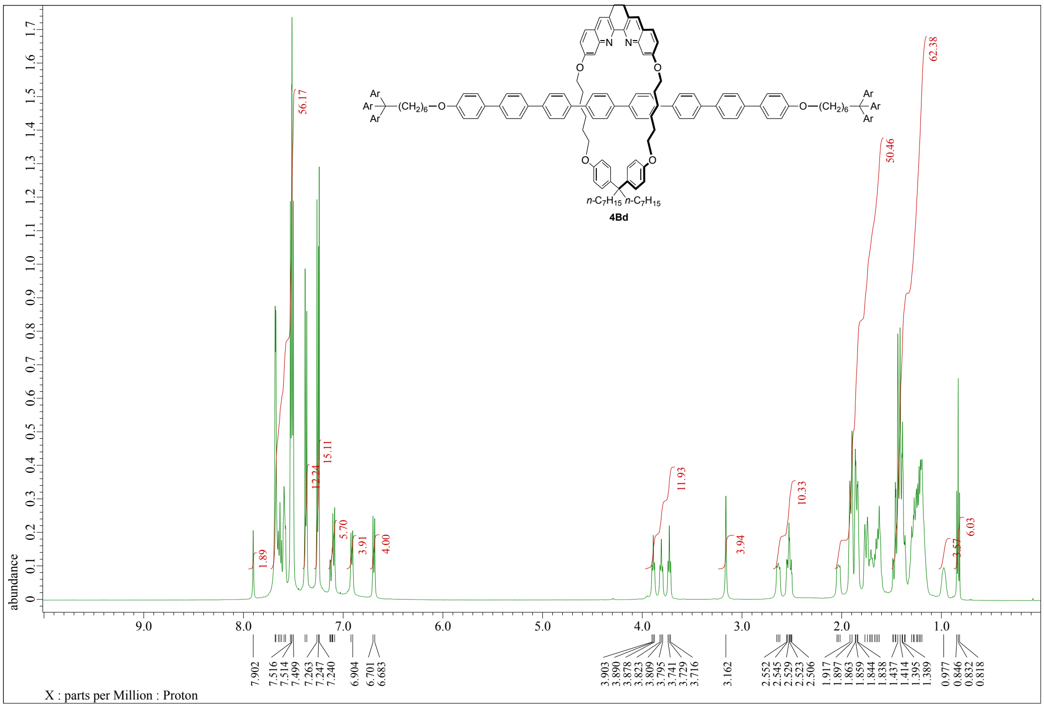

^{1}H NMR spectrum of **4Bd** (500 MHz, $CDCl_3$)

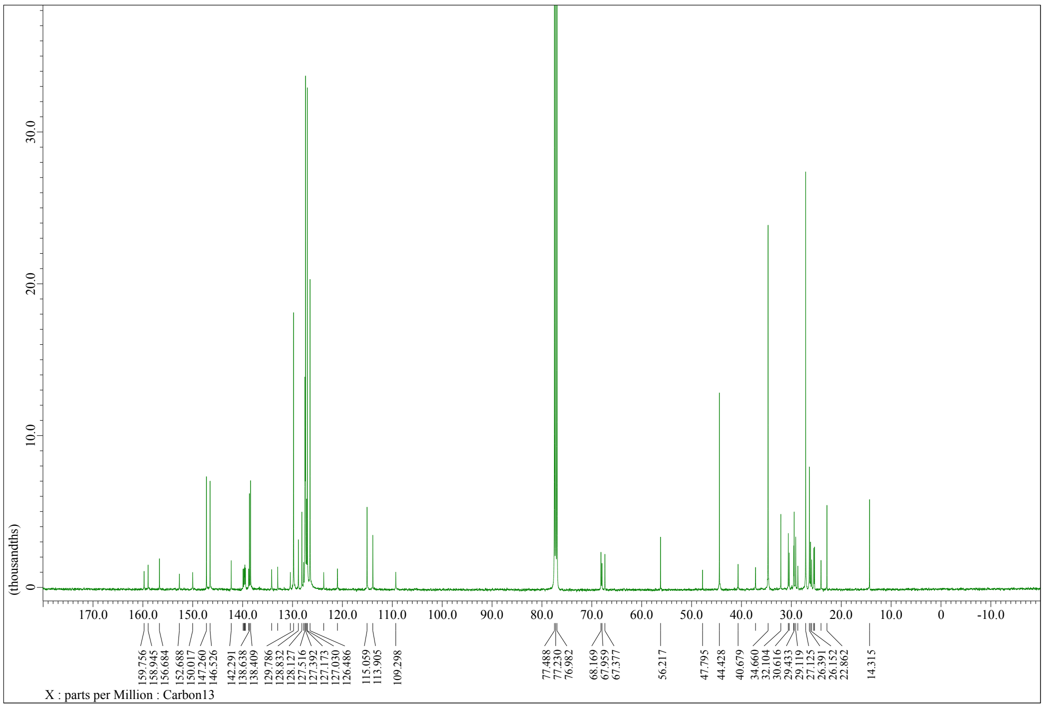

^{13}C NMR spectrum of **4Bd** (125 MHz, $CDCl_3$)

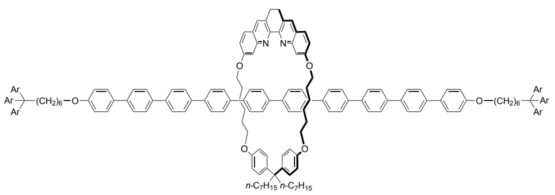

4Be

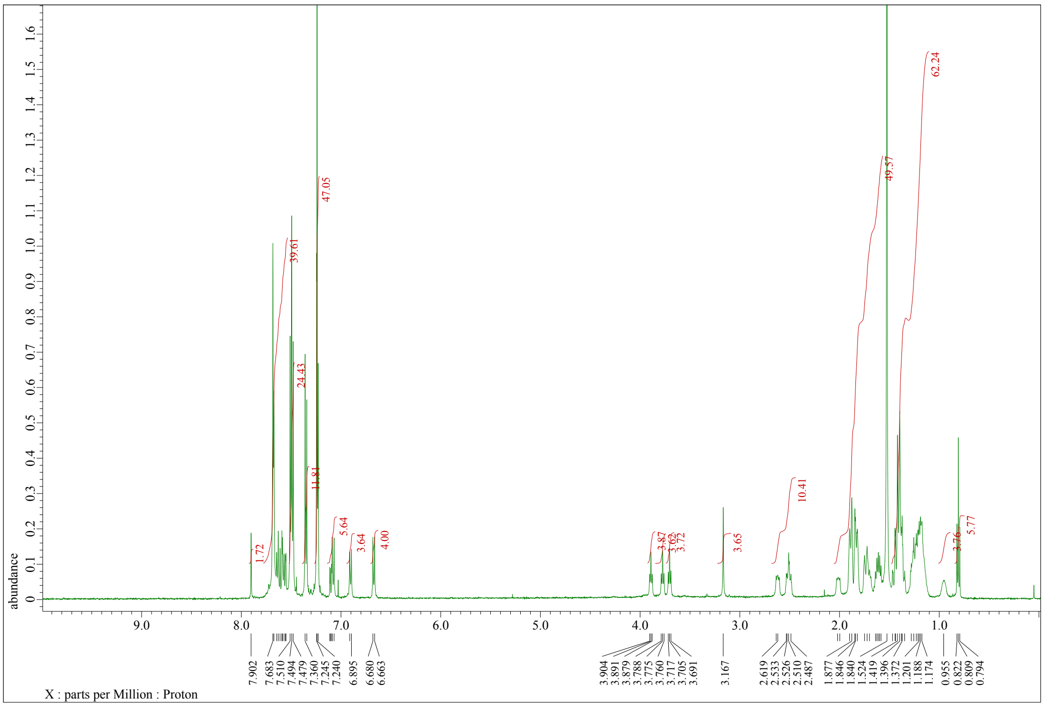

^{1}H NMR spectrum of **4Be** (500 MHz, $CDCl_3$)

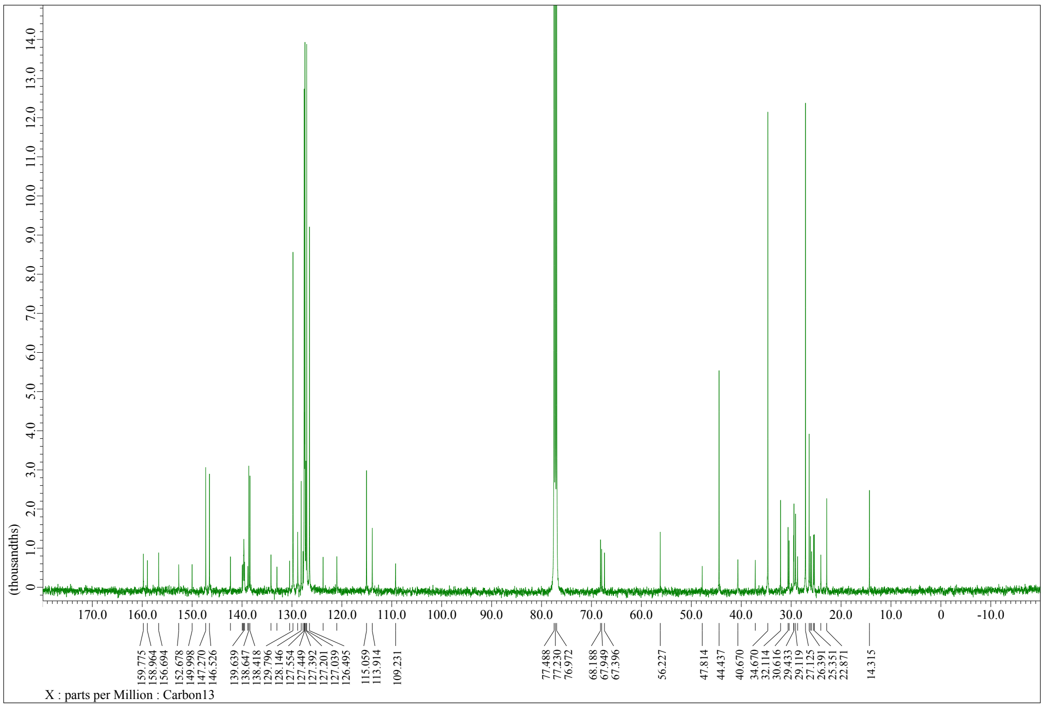

^{13}C NMR spectrum of **4Be** (125 MHz, $CDCl_3$)

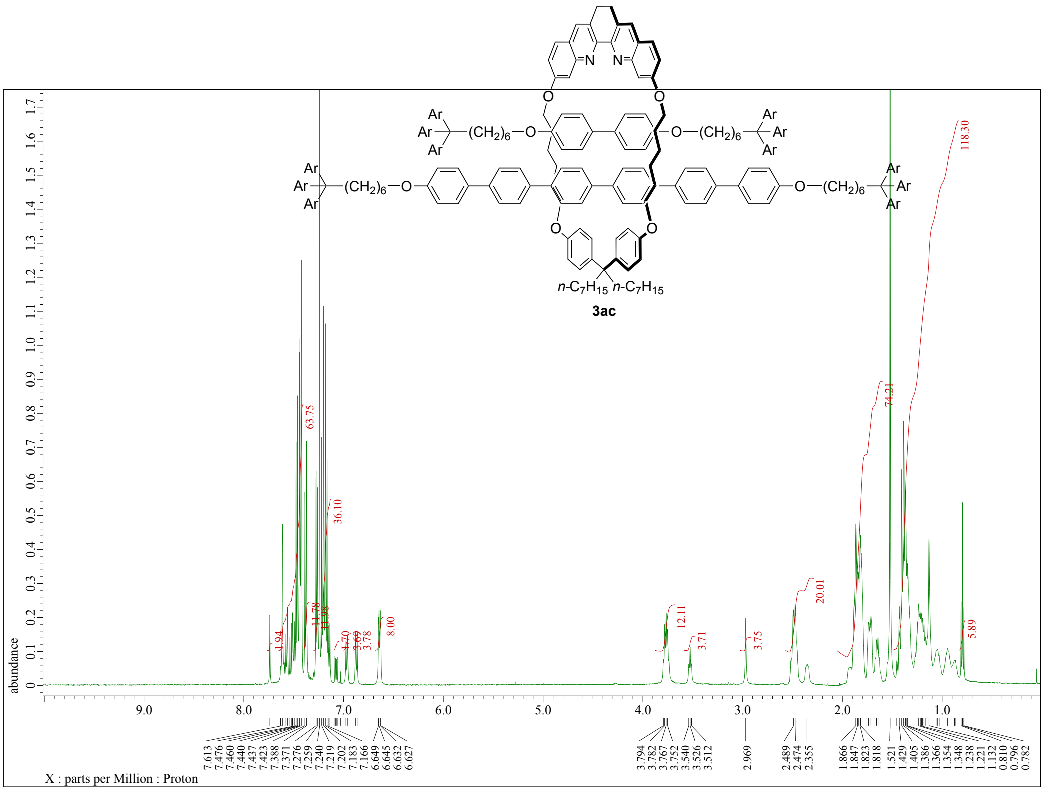

^{1}H NMR spectrum of **3ac** (500 MHz, $CDCl_3$)

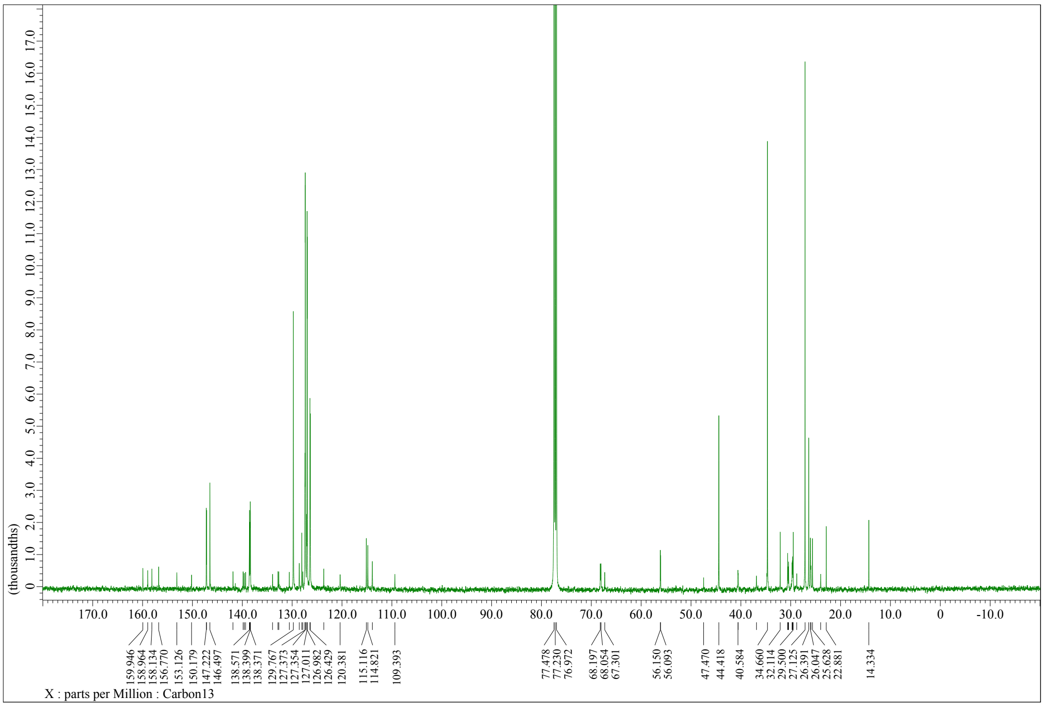

^{13}C NMR spectrum of **3ac** (125 MHz, $CDCl_3$)

4. Details of GPC analysis

The recycle GPC analysis (Fig. 4) was done by using following conditions.

Column : YMC-GPC T30000 (21.2 mm ID × 600 mm L)
Eluent : $CHCl_3$
Flow rate : 10 mL/min
Detection : 254 nm

1.0 mL of a 2.0 mM solution of the sample was injected in Fig. 4a, while 1.0 mL of a 10 mM solution of the sample was injected in Fig. 4b. The samples were recycled once.

5. DLS and SLS analysis

5.1 Details of DLS analysis

DLS (Dynamic Light Scattering) measurement was conducted at 25 °C in chloroform. Measurements were taken at concentrations of 4.0, 2.0, 1.0, 0.50, and 0.25 mM, at angles of 30°, 60°, 90°, 120°, and 150°. Data points were trimmed within a standard deviation range of ±0.03, and exponential fittings were conducted. The peak of the largest area was selected as the data point. The DLS data at 150° which is the angle with the least noise were plotted in Fig. 5, and values from other angles represented as error bars.

5.2 Autocorrelation function

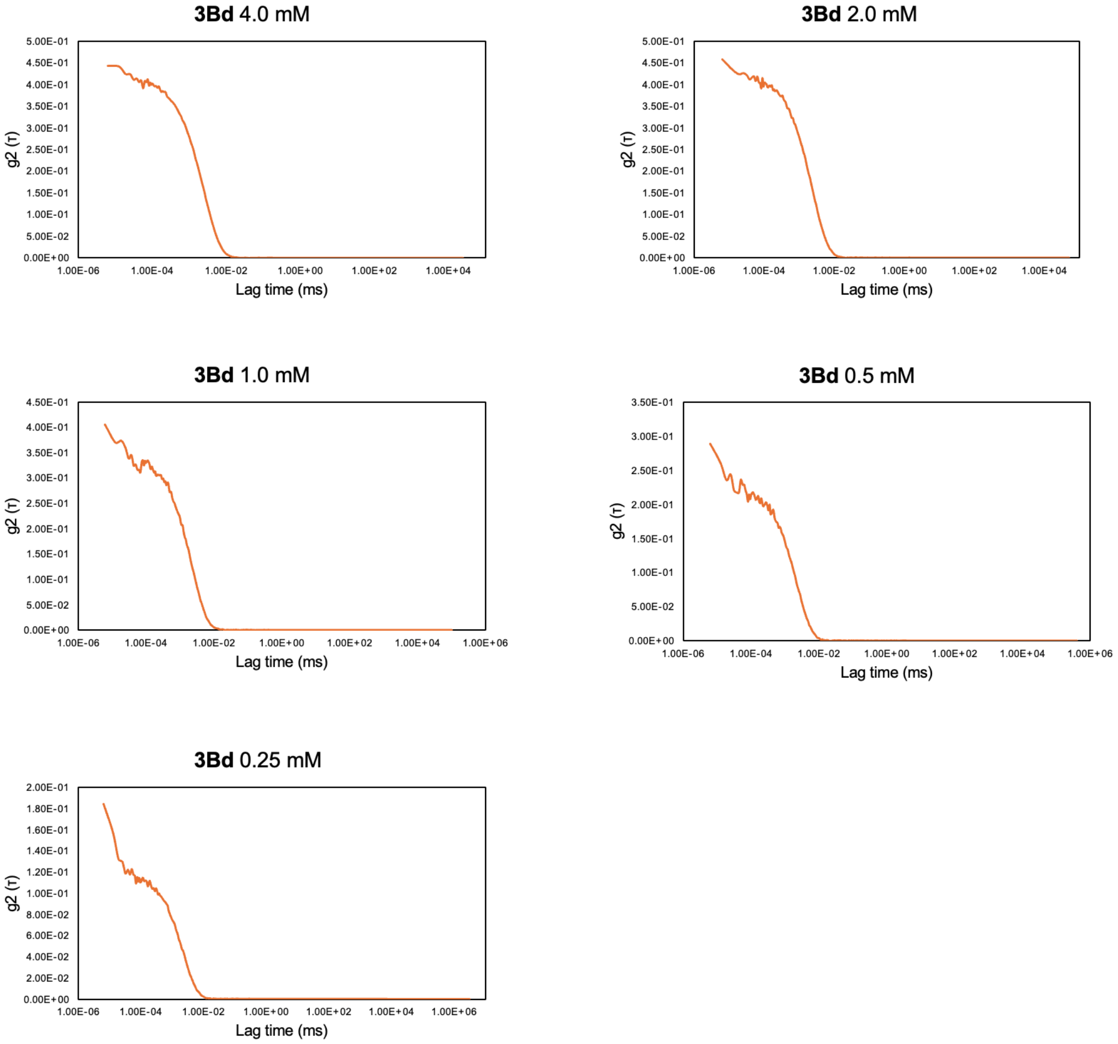

Autocorrelation functions of **3Bd** in 4.0, 2.0, 1.0, 0.5 and 0.25 mM at 150 °.

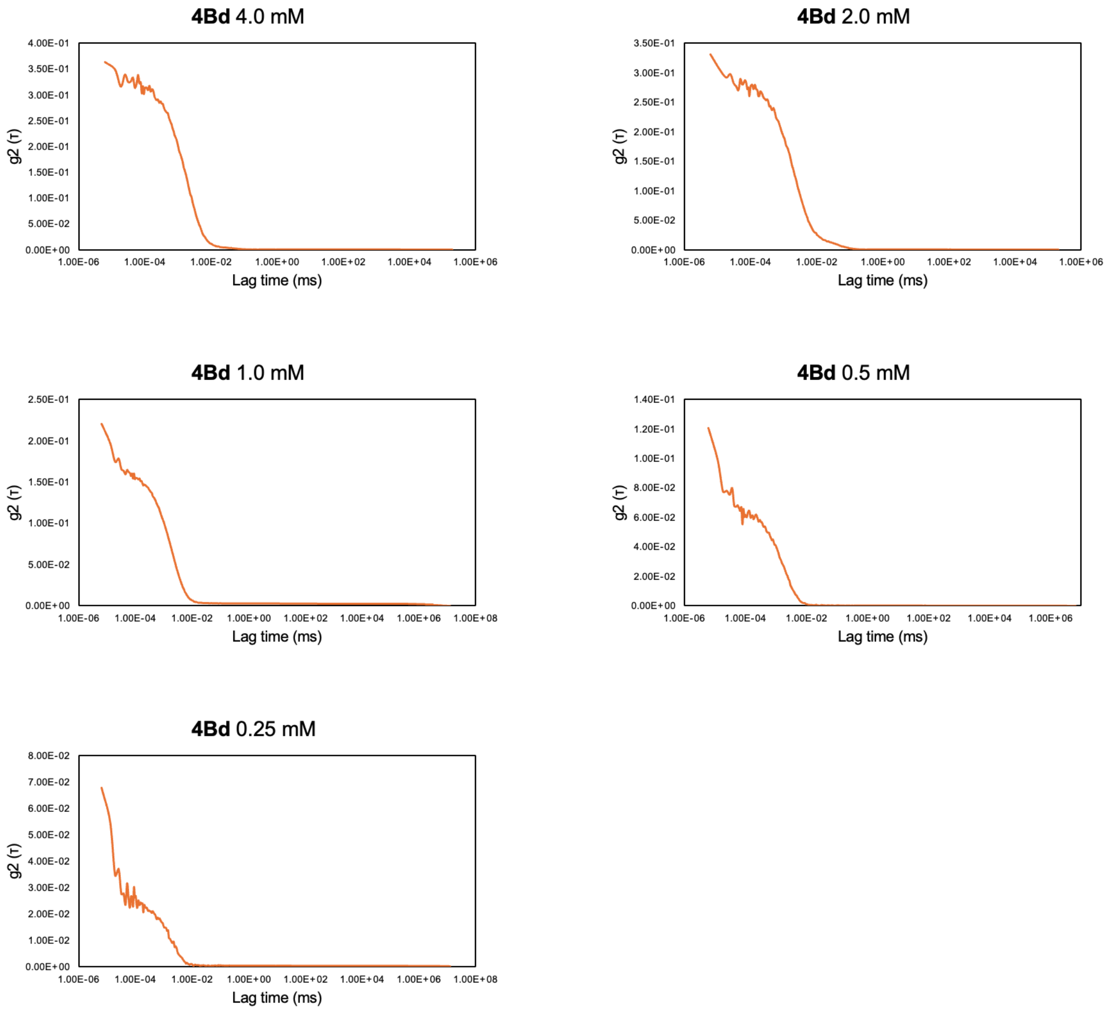

Autocorrelation functions of **4Bd** in 4.0, 2.0, 1.0, 0.5 and 0.25 mM at 150 °.

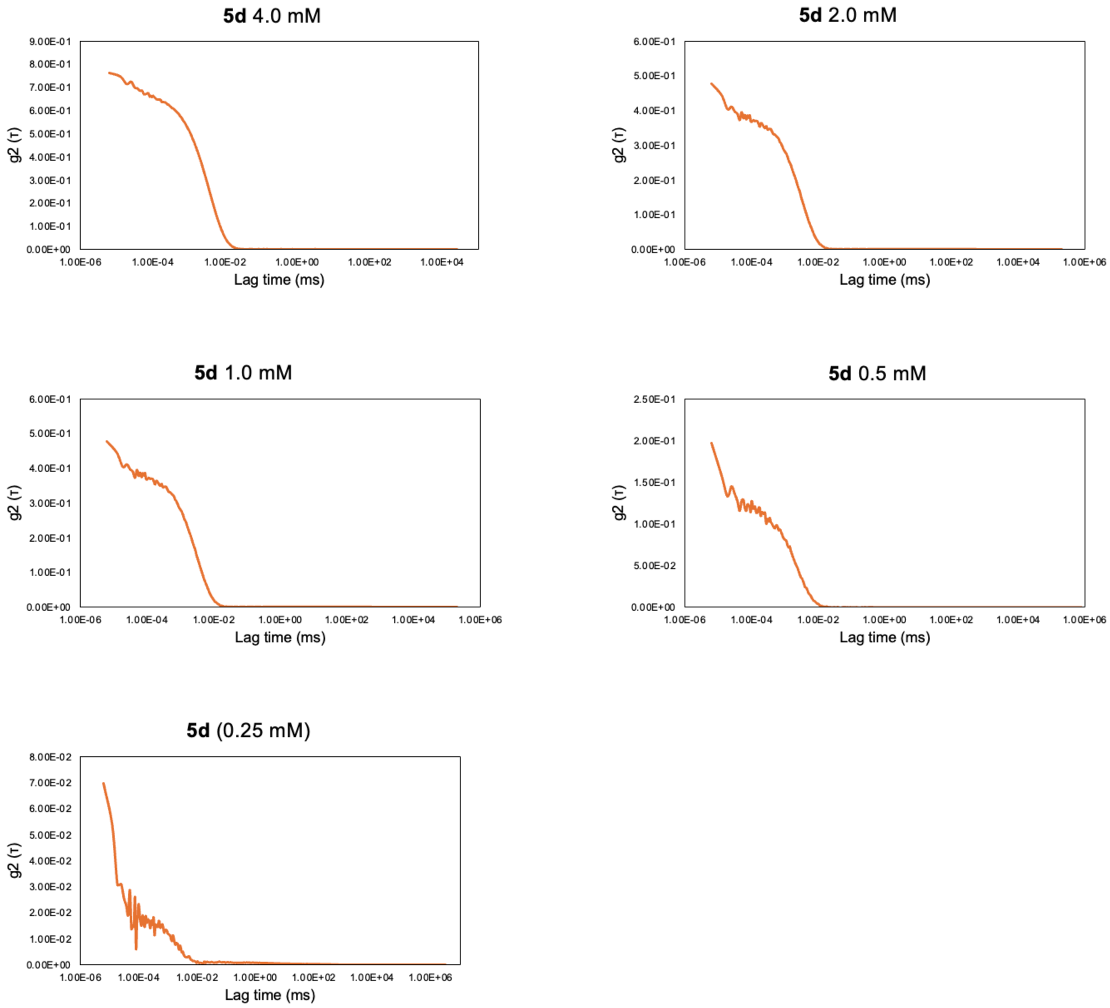

Autocorrelation functions of **5d** in 4.0, 2.0, 1.0, 0.5 and 0.25 mM at 150 °.

5.3 SLS analysis of **5d**, **4Bd**, and **3Bd**

The weight-average molecular weight (Mw) of **5d**, **4Bd**, and **3Bd** were obtained by SLS measurement, and the results are summarized in Fig. S1. Similar results were obtained in SLS as in DLS. The calculated Mw of **5d** increased as the concentration of **5d** increased, indicating the formation of the aggregate. In contrast, the calculated Mw of rotaxanes remained constant. The concentration dependence of **5d** differed between SLS and DLS, with SLS showing a more gradual increase. This would be due to differences observed physical properties (Rh vs. Mw). Since Rh is more strongly influenced by larger molecules than Mw, the increase in Rh is expected to be more pronounced.

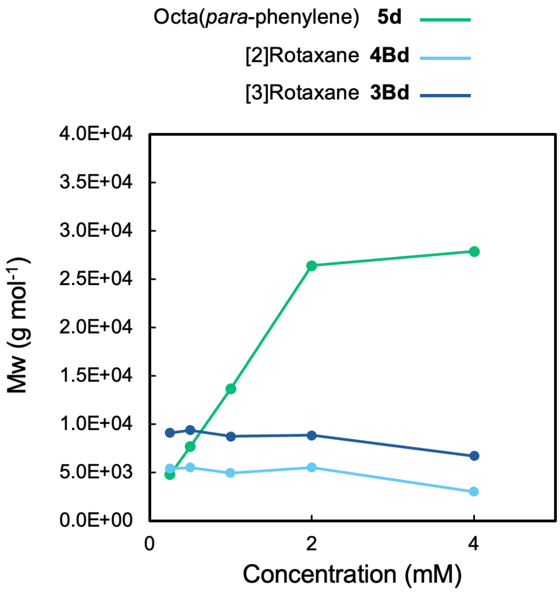

Figure S1. The Mw of **5d**, **4Bd**, and **3Bd** in 4.0, 2.0, 1.0, 0.50, 0.25 mM by SLS.

Second virial coefficients (A2) of **5d**, **4Bd**, and **3Bd** were as follows.

$$A2\ (\mathbf{5d}) = -8.25\times10^{-3}\ \mathrm{mol\ cm^{3}\ g^{-2}}$$
$$A2\ (\mathbf{4Bd}) = 5.96\times10^{-3}\ \mathrm{mol\ cm^{3}\ g^{-2}}$$
$$A2\ (\mathbf{3Bd}) = 9.88\times10^{-4}\ \mathrm{mol\ cm^{3}\ g^{-2}}$$

When A2 is negative, attractive forces exist between the particles in the solution. When A2 is positive, repulsive forces are present. In this case, only A2 (**5d**) was negative, indicating the presence of attractive forces between the molecules of **5d**. These results are consistent with the data obtained by other measurements.
